# Reconvalescent plasma/camostat mesylate in early SARS-CoV-2 Q-PCR positive high-risk individuals (RES-Q-HR): a structured summary of a study protocol for a randomized controlled trial

**DOI:** 10.1186/s13063-021-05181-0

**Published:** 2021-05-17

**Authors:** Verena Keitel, Björn Jensen, Torsten Feldt, Johannes C. Fischer, Johannes G. Bode, Christiane Matuschek, Edwin Bölke, Wilfried Budach, Christian Plettenberg, Kathrin Scheckenbach, Detlef Kindgen-Milles, Jörg Timm, Lisa Müller, Henrike Kolbe, Andreas Stöhr, Christian Calles, Andreas Hippe, Pablo Verde, Christoph D. Spinner, Jochen Schneider, Timo Wolf, Winfried V. Kern, Jacob Nattermann, Alexander Zoufaly, Christian Ohmann, Tom Luedde, RES-Q-HR Trial Team, Simon Labuhn, Noemi Freise, Alexander Killer, Caroline Klindt, Carola Dröge, Anselm Kunstein, David Schoeler, Sandra Jost, Erik Lehnert, Stefanie Ackerstaff, Timo Brandenburger, Christina Westhoff, Christine Fritsch, Stephanie Laer, Andrea Icks

**Affiliations:** 1 Department of Gastroenterology, Hepatology and Infectious Diseases, University Hospital Duesseldorf, Medical Faculty Heinrich-Heine-University Duesseldorf, Moorenstr. 5, D-40225 Duesseldorf, Germany; 2 Institute for Transplant Diagnostics and Cell Therapeutics, University Hospital Duesseldorf, Medical Faculty Heinrich-Heine-University Duesseldorf, Moorenstr. 5, D-40225 Duesseldorf, Germany; 3 Department of Radiation Oncology, University Hospital Duesseldorf, Medical Faculty, Heinrich-Heine-University Duesseldorf, Moorenstr. 5, D-40225 Duesseldorf, Germany; 4 Department of Ear, Nose and Throat disease, University Hospital Duesseldorf, Medical Faculty Heinrich-Heine-University Duesseldorf, Moorenstr. 5, D-40225 Duesseldorf, Germany; 5 Department of Anesthesiology, University Hospital Duesseldorf, Medical Faculty Heinrich-Heine-University Duesseldorf, Moorenstr. 5, D-40225 Duesseldorf, Germany; 6 Institute for Virology, University Hospital Duesseldorf, Medical Faculty Heinrich-Heine-University Duesseldorf, Universitaetsstr. 1, D-40225 Duesseldorf, Germany; 7 Coordination Center for Clinical Studies, Medical Faculty, Heinrich Heine University Duesseldorf, D-40225 Duesseldorf, Germany; 8 Technical University of Munich, School of Medicine, University Hospital Rechts der Isar, Ismaninger Str. 22, D-81675 Munich, Germany; 9 Department of Internal Medicine, Infectious Diseases, University Hospital Frankfurt, Goethe University, Theodor-Stern-Kai 7, D-60590 Frankfurt am Main, Germany; 10 Department of Internal Medicine II, Infectious Diseases, University Hospital Freiburg, Hugstetter Str. 55, D-79106 Freiburg, Germany; 11 Department of Internal Medicine I, University Hospital Bonn, Sigmund-Freud-Str. 25, D-53127 Bonn, Germany; 12 Department of Medicine IV, Kaiser Franz Josef Hospital, Kundratstrasse 3, 1100 Vienna, Austria

**Keywords:** COVID-19, Randomized controlled trial, Protocol, Convalescent plasma, Camostat mesylate, Antiviral therapy, Early phase of SARS-CoV-2 infection

## Abstract

**Objectives:**

Currently, there are no approved treatments for early disease stages of COVID-19 and few strategies to prevent disease progression after infection with SARS-CoV-2. The objective of this study is to evaluate the safety and efficacy of convalescent plasma (CP) or camostat mesylate administered within 72 h of diagnosis of SARS-CoV-2 infection in adult individuals with pre-existing risk factors at higher risk of getting seriously ill with COVID-19. Camostat mesylate acts as an inhibitor of the host cell serine protease TMPRSS2 and prevents the virus from entering the cell. CP represents another antiviral strategy in terms of passive immunization. The working hypothesis to be tested in the RES-Q-HR study is that the early use of CP or camostat mesylate reduces the likelihood of disease progression to (modified) WHO stages 4b-8 in SARS-CoV-2-positive adult patients at high risk of moderate or severe COVID-19 progression.

**Trial design:**

This study is a 4-arm (parallel group), multicenter, randomized (2:2:1:1 ratio), partly double-blind, controlled trial to evaluate the safety and efficacy of convalescent plasma (CP) or camostat mesylate with control or placebo in adult patients diagnosed with SARS-CoV-2 infection and high risk for progression to moderate/severe COVID-19. Superiority of the intervention arms will be tested.

**Participants:**

The trial is conducted at 10–15 tertiary care centers in Germany. Individuals aged 18 years or above with ability to provide written informed consent with SARS-CoV-2 infection, confirmed by PCR within 3 days or less before enrolment and the presence of at least one SARS-CoV-2 symptom (such as fever, cough, shortness of breath, sore throat, headache, fatigue, smell/and or taste disorder, diarrhea, abdominal symptoms, exanthema) and symptom duration of not more than 3 days.

Further inclusion criteria comprise:

Presence of at least one of the following criteria indicating increased risk for severe COVID-19:

- Age > 75 years
- Chronic obstructive pulmonary disease (COPD) and/or pulmonary fibrosis
- BMI > 40 kg/m^2^
- Age > 65 years with at least one other risk factor (BMI > 35 kg/m^2^, coronary artery disease (CAD), chronic kidney disease (CKD) with GFR < 60 ml/min but ≥ 30 ml/min, diabetes mellitus, active tumor disease)
- BMI > 35 kg/m^2^ with at least one other risk factor (CAD, CKD with GFR < 60 ml/min but ≥ 30 ml/min, diabetes mellitus, active tumor disease)

Exclusion criteria:

1. Age < 18 years
2. Unable to give informed consent
3. Pregnant women or breastfeeding mothers
4. Previous transfusion reaction or other contraindication to a plasma transfusion
5. Known hypersensitivity to camostat mesylate and/or severe pancreatitis
6. Volume stress due to CP administration would be intolerable
7. Known IgA deficiency
8. Life expectancy < 6 months
9. Duration SARS-CoV-2 typical symptoms > 3 days
10. SARS-CoV-2 PCR detection older than 3 days
11. SARS-CoV-2 associated clinical condition ≥ WHO stage 3 (patients hospitalized for other reasons than COVID-19 may be included if they fulfill all inclusion and none of the exclusion criteria)
12. Previously or currently hospitalized due to SARS-CoV-2
13. Previous antiviral therapy for SARS-CoV-2
14. ALT or AST > 5 x ULN at screening
15. Liver cirrhosis > Child A (patients with Child B/C cirrhosis are excluded from the trial)
16. Chronic kidney disease with GFR < 30 ml/min
17. Concurrent or planned anticancer treatment during trial period
18. Accommodation in an institution due to legal orders (§40(4) AMG).
19. Any psycho-social condition hampering compliance with the study protocol.
20. Evidence of current drug or alcohol abuse
21. Use of other investigational treatment within 5 half-lives of enrolment is prohibited
22. Previous use of convalescent plasma for COVID-19
23. Concomitant proven influenza A infection
24. Patients with organ or bone marrow transplant in the three months prior to screening visit

**Intervention and comparator:**

Participants will be randomized to the following 4 groups:

1. Convalescent plasma (CP), 2 units at screening/baseline visit (day 0) or day 1; CP is defined by the presence of neutralizing anti-SARS-CoV-2 antibodies with titers ≥ 1:160; individuals with body weight ≥ 150 kg will receive a third unit of plasma on day 3
2. Camostat mesylate (200 mg per capsule, one capsule taken each in the morning, afternoon and evening on days 1–7)
3. Standard of care (SOC, control for CP)
4. Placebo (identical in appearance to camostat mesylate capsules, one capsule taken each morning, afternoon and evening on days 1–7; for camostat mesylate control group)

Participants will be monitored after screening/baseline on day 3, day 5, day 8, and day 14. On day 28 and day 56, telephone visits and on day 90, another outpatient visit are scheduled.

Adverse events and serious adverse events will be monitored and reported until the end of the study. An independent data safety monitoring committee will review trial progression and safety.

**Main outcomes:**

The primary endpoint of the study is the cumulative number of individuals who progress to or beyond category 4b on the modified WHO COVID-19 ordinal scale (defined as hospitalization with COVID-19 pneumonia and additional oxygen demand via nasal cannula or mask) within 28 days after randomization.

**Randomization:**

Participants will be randomized using the Alea-Tool (aleaclinical.com) in a 2:2:1:1 ratio to the treatment arms (1) CP, (2) camostat mesylate, (3) standard of care (SoC), and (4) placebo matching camostat mesylate. Randomization will be stratified by study center.

**Blinding (masking):**

The camostat mesylate treatment arm and the respective placebo will be blinded for participants, caregivers, and those assessing outcomes.

The treatment arms convalescent plasma and standard of care will not be blinded and thus are open-labeled, unblinded.

**Numbers to be randomized (sample size):**

Overall, *n* = 994 participants will be randomized to the following groups: *n* = 331 to convalescent plasma (CP), *n* = 331 to camostat mesylate, *n* = 166 to standard of care (SoC), and *n* = 166 to placebo matching camostat mesylate.

**Trial status:**

The RES-Q-HR protocol (V04F) was approved on the 18 December 2020 by the local ethics committee and by the regulatory institutions PEI/BfARM on the 2 December 2020. The trial was opened for recruitment on 26 December 2020; the first patient was enrolled on 7 January 2021 and randomized on 8 January 2021.

Recruitment shall be completed by June 2021. The current protocol version RES-Q HR V05F is from 4 January 2021, which was approved on the 18 January 2021.

**Trial registration:**

EudraCT Number 2020-004695-18. Registered on September 29, 2020.

ClinicalTrial.gov NCT04681430. Registered on December 23, 2020, prior to the start of the enrollment (which was opened on December 26, 2020).

**Full protocol:**

The full protocol (V05F) is attached as an additional file, accessible from the Trials website (Additional file 1). In the interest in expediting dissemination of this material, the familiar formatting has been eliminated; this letter serves as a summary of the key elements of the full protocol.

The study protocol has been reported in accordance with the Standard Protocol Items: Recommendations for Clinical Interventional Trials (SPIRIT) guidelines (Additional file 2).

**Supplementary Information:**

The online version contains supplementary material available at 10.1186/s13063-021-05181-0.

## Supplementary Information

**Additional file 1.** Full study protocol.

**Additional file 2.** SPIRIT 2013 Checklist: Recommended items to address in a clinical trial protocol and related documents*.

## Data Availability

All investigators will have access to the final trial dataset. Data will be available from the corresponding author upon request.

