# Supplementary material for: Reconvalescent plasma/camostat mesylate in early SARS-CoV-2 Q-PCR positive high-risk individuals (RES-Q-HR): a structured summary of a study protocol for a randomized controlled trial
Source: Trials. 2021 May 17;22:343. doi: 10.1186/s13063-021-05181-0 (PMC8127198; doi:10.1186/s13063-021-05181-0)
Supplement: Supplementary file 1 — Additional file 1. Full study protocol. [file 13063_2021_5181_MOESM1_ESM.pdf]

Reconvalescent plasma/Camostat mesylate early in SARS-CoV-2 Q-PCR positive high-risk individuals

Protocol

Protocol code / Acronym

RES-Q HR

EudraCT-Number:

2020-004695-18

Sponsor of the clinical study

Heinrich-Heine-University Düsseldorf
Universitätsstraße 1
D-40225 Düsseldorf

Principal Coordinating Investigators

Prof. Dr. med. Verena Keitel-Anselmino

PD Dr. med. Torsten Feldt

Dr. Björn-Erik Ole Jensen

Steering Committee

Prof. Dr. Edwin Bölke

Prof. Dr. Johannes Bode

Prof. Dr. Dr. Tom Lüdde

Version V05F / 04 Jan 2021

- Confidential -

The information contained in this protocol has to be kept strictly confidential. Therefore, the protocol is only provided to Investigators in confidence for review, to study staff, Independent Ethics Committee/Institutional Review Board, regulatory authorities and CROs (or CTO) and for obtaining written informed consent from patients.

Protocol adopted from the master protocol “A Multi-center, Adaptive, Randomized Blinded Controlled Trial of the Safety and Efficacy of Investigational Therapeutics for the Treatment of COVID-19 in Hospitalized Patients” by Dr. U. Behrens, Dr. A. Krannich & Prof. Dr. C. von Kalle

Sponsor Signature Page

Sponsor:	[signature]	5.1.21
	Signature of Sponsor's Representative	Date
	Verena Keitel-Anselmino	
	Printed Name of Sponsor's Representative	
	By my signature, I agree to supervise the conduct of this study and to ensure its conduct in compliance with the protocol, informed consent, IRB/EC procedures, the Declaration of Helsinki, ICH Good Clinical Practices guidelines, and local regulations governing the conduct of clinical studies.	

Principal Coordinating Investigator Signature Page

Principal Coordinating Investigator:	[signature]	5.1.21
	Signature of Principal Coordinating Investigator	Date
	Verena Keitel-Anselmino	
	Printed Name of Coordinating Investigator	
	By my signature, I agree to personally supervise the conduct of this study and to ensure its conduct in compliance with the protocol, informed consent, IRB/EC procedures, the Declaration of Helsinki, ICH Good Clinical Practices guidelines, and local regulations governing the conduct of clinical studies.	

Statistician Signature Page

Statistician:		
	6.01.2021	
	Signature of Statistician	Date
	Pablo E. Verde	
	Printed name of Statistician	

Table of contents

ABBREVIATIONS 11

SYNOPSIS 14

1 INTRODUCTION 22

1.1 Background and Purpose of the Study 22

1.2 Study Design 23

1.3 Study Scheme 24

1.4 Planned Intervention and Selection of Study Population 25

2 RISK/BENEFIT ASSESSMENT FOR THE STUDY 26

2.1 Known Potential Risks 26

2.2 Potential Side Effects of the Study Medication 27

2.2.1 Camostat Mesylate 27

2.2.2 Convalescent Plasma 28

2.3 Potential Risks of Having Blood Drawn 29

2.4 Potential Risks of Nasopharyngeal Swab 30

2.5 Risks to Privacy 30

2.6 Known Potential Benefits 30

2.7 Assessment of Potential Risks and Benefits 31

3 ENDPOINTS AND STUDY DESIGN 31

3.1 Endpoints 31

3.1.1 Primary Study Endpoint 31

3.1.2 Secondary Study Endpoints 32

3.2 Study Design 32

3.2.1 Overall Design 32

3.2.2 Justification for Dose 33

3.3 Timetable of the Study 33

4 STUDY POPULATION 34

4.1 Study Population Selection 34

4.2 Inclusion Criteria 34

4.3 Exclusion Criteria 36

4.4 Screening Failures 37

4.5 Strategies for Recruitment and Retention 37
4.5.1 Recruitment 37
4.5.2 Retention 37
4.5.3 Compensation Plan for Subjects 37
4.5.4 Costs 37

5 INVESTIGATIONAL MEDICINAL PRODUCTS 38
5.1 Convalescent Plasma 38
5.1.1 Description and Administration 38
5.1.2 Labelling of CP 40
5.1.3 Dosing and Administration of CP 40
5.1.4 Allocation and Transfusion of CP from a Donor to a Study Participant 41
5.1.5 Accountability, Product Storage, Stability and Destruction 43
5.1.6 Product Storage, Stability and Destruction 43
5.2 Camostat Mesylate 43
5.2.1 Description 43
5.2.2 Formulation, Labelling and Packaging of Camostat Mesylate and Matching Placebo 44
Camostat Mesylate 44
Matching Placebo 44
5.2.3 Dosing and Administration 44
5.2.4 Accountability, Product Storage, Stability and Destruction 44
5.3 Treatment Compliance 45
5.4 Concomitant Medication / Concomitant Therapy 45
5.5 Rescue Medicine and Procedures in Case of Emergency 46
5.6 Blinding Procedures and Unblinding 46

6 STUDY PROCEDURES 46
6.1 Recruitment / Screening Procedures 46
6.2 Methods of Obtaining Informed Consent 46
6.3 Methods of Avoiding Simultaneous Enrolment in Other Trials 46
6.4 Enrolment and Randomisation (Assignment of Study Medication) 47
6.5 Measures to Minimize Bias: Randomization and Blinding 47
6.6 Clinical Examinations and Trial-related Deviations from Clinical Practice 47
6.6.1 Pre-trial Examinations (Screening / Inclusion Examination) 47
6.6.2 Examinations During Trial 48
6.6.3 Final Examination (day 90 ± 5 days) 49

6.6.4 Exploratory Assessments (optional) ... 50

7 DISCONTINUATION AND WITHDRAWAL ... 50

7.1 Individual Halting ... 50

7.1.1 Premature Termination of the Clinical Study ... 51

7.2 Lost to Follow-Up ... 52

7.3 Follow-up and Continuing Treatment after Regular Termination ... 52

8 SAFETY ... 52

8.1 Definition of Adverse Events and Serious Adverse Events ... 52

8.1.1 Adverse Event and Adverse Drug Reaction ... 52

8.1.2 Serious Adverse Event and Serious Adverse Drug Reaction ... 53

8.1.3 Suspected Unexpected Serious Adverse Reactions ... 53

8.2 Severity / Intensity ... 53

8.3 Causality ... 53

8.4 Documentation and follow-up of AEs and SAEs ... 54

8.5 Reporting of Serious Adverse Events, pregnancies and changes in risk-benefit assessment.. 55

8.5.1 Notification of the Sponsor by the Investigator ... 55

8.5.2 Assessment of the Serious Adverse Events by the Sponsor ... 55

8.5.3 Notification of National Competent Authority, Leading Ethics Committee, and Trial Sites 56

8.5.4 Evaluation and Report of Changes in Risk/Benefit Ratio ... 56

8.6 Pregnancies and Investigator Reporting Responsibilities ... 56

8.6.1 Female of Childbearing Potential ... 56

8.6.2 Male Subject ... 57

8.7 Development Safety Update Report ... 57

8.8 Data Safety Monitoring Committee ... 57

9 QUALITY MANAGEMENT ... 58

9.1 Principal Investigator Responsibilities ... 58

9.2 Quality Assurance and Quality control ... 58

9.3 Study Monitoring ... 58

9.4 Audits ... 59

9.5 Source Data and Patient Files ... 59

9.6 Recording, Analyses and Reporting ... 59

9.7 Data Collection ... 60

10 STATISTICAL ANALYSIS ... 61

10.1 Statistical Hypotheses ... 61
10.2 Sample Size Determination ... 61
10.3 Populations for Analyses ... 61
10.4 Statistical Analyses ... 61
10.4.1 Baseline descriptive statistics ... 61
10.4.2 Analysis of the Primary Efficacy Endpoint ... 61
10.4.3 Analysis of the Secondary Endpoint(s) ... 62
10.4.4 Safety Analyses ... 62
10.4.5 Planned Interim Analyses ... 63
10.4.6 Sub-Group Analyses ... 64
10.4.7 Exploratory Analyses ... 64
10.5 Reporting ... 64
10.5.1 Statistical Report ... 64
10.5.2 Final Report ... 64
10.5.3 Publication Policy ... 64

11 ETHICAL, LEGAL AND REGULATORY ASPECTS ... 65
11.1 ICH-GCP-Guidelines ... 65
11.2 Legal Requirements of the Study ... 65
11.2.1 Approval of Ethics Committee ... 65
11.2.2 Approval of national competent Authorities ... 65
11.2.3 Notification to Local Authorities ... 65
11.3 Patient Information and Informed Consent ... 65
11.4 Patient Insurance ... 66
11.5 Data Privacy and Confidentiality ... 66
11.6 Archiving of Data / Access to Records ... 66

12 FINANCING ... 66

13 REFERENCES ... 67

14 APPENDICES ... 70
14.1 Schedule of Events ... 70
14.2 Visual Analogue Scale (VAS) ... 72
14.3 Data Safety and Monitoring Board DSMB ... 73
14.4 Quality of life questionnaire (SF-12) ... 73

List of tables

Table 1 Definition of risk groups 34

Table 2 Collection volume per plasmapheresis and resulting number and volume of therapeutic units 38

Table 3 Transfusion therapy with CP in SARS-CoV-2: selection of the ABO phenotype of units to transfuse 42

List of figures

Figure 1 RES-Q HR Flow Chart 25

ABBREVIATIONS

ADE	Antibody-mediated enhancement
ADR	Adverse Drug Reaction
AE	Adverse Event
ACE	Angiotensin Converting Enzyme
ALP	Alkaline Phosphatase
ALT	Alanine Aminotransferase
AMG	Arzneimittelgesetz
ARDS	Acute Respiratory Distress Syndrome
AST	Aspartate Aminotransferase
BfArM	Das Bundesinstitut für Arzneimittel und Medizinprodukte
BMG	Bundesministerium für Gesundheit
BMI	Body Mass Index
BUN	Blood Urea Nitrogen
CAD	Coronary Artery Disease
CFR	Case Fatality Rate
CK	Creatine Kinase
CKD	Chronic Kidney Disease
COPD	Chronic Obstructive Pulmonary Disease
COVID-19	Coronavirus Disease
CP	Convalescent plasma
Crea	Creatinine
CRF	Case Report Form
DSMB	Data Safety and Monitoring Board
DSUR	Development Safety Update Report
EC	Ethics Committee
EC_{50}	Half maximal effective concentration
ECMO	Extracorporeal Membrane Oxygenation
EOT	End of Treatment
EUA	Emergency Use Authorization
FDA	Food and Drug Administration
FFP	Fresh Frozen Plasma
GCP	Good Clinical Practice
GFR	Glomerular Filtration Rate
GGT	Gamma Glutamyl Transferase
HBV	Hepatitis B virus
HCIP	Human Coronavirus Immune Plasma

HCV	Hepatitis C virus
HIV	Human immunodeficiency virus
IB	Investigator's Brochure
ICF	Informed Consent Form
ICH	International Council for Harmonisation
ICU	Intensive Care Unit
IEC	Independent Ethics Committee
IMC	Intermediate Care Unit
INR	International Normalized Ratio
IRB	Institutional Review Board
IRF	Infection Fatality Rate
ITT	Intention to Treat Population
ITZ	Institut für Transplantationsdiagnostik und Zelltherapeutika
IV	Intravenous
KKSD	Koordinierungszentrum für Klinische Studien Düsseldorf
MAR	Missing at Random
MedDRA	Medical Dictionary for Regulatory Activities
MERS	Middle East Respiratory Syndrome
miRNA	Micro Ribonucleid Acid
MOP	Manual of Procedures
N	Number (typically refers to subjects)
NIH	National Institutes of Health
NMAR	Not Missing at Random
NP	Nasopharyngeal
NT	Neutralizing (antibody) Titer
NT-proBNP	N- terminal pro brain natriuretic peptide
OR	Odds Ratio
PCI	Principal Coordinating Investigator
PCT	Procalcitonin
PHI	Protected/Personal Health Information
PI	Principal Investigator
PT	Prothrombin Time
RKI	Robert-Koch Institut
SAE	Serious Adverse Event
SAP	Statistical Analysis Plan
SARS	Severe Acute Respiratory Syndrome
SARS-CoV-2	Severe Acute Respiratory Syndrome Coronavirus 2
SDV	Source Data Verification
SoC	Standard of Care
SOP	Standard Operating Procedure

STAKOB	Ständiger Arbeitskreis der Kompetenz- und Behandlungszentren für Krankheiten durch hochpathogene Erreger
SuPAR	Soluble Urokinase-type Plasminogen Activator Receptor
SUSAR	Suspected Unexpected Serious Adverse Reaction
TACO	Transfusion-associated circulatory overload
TMPRSS2	Transmembrane Serine Protease 2
TRALI	Transfusion-related acute lung injury
UK	United Kingdom
ULN	Upper Limit of Normal
VAS	Visual Analogue Scale
WNV	West Nile Virus

SYNOPSIS

**Study title**	Reconvalescent plasma / Camostat mesylate early in Sars-CoV-2 Q-PCR positive high-risk individuals
**EudraCT Number**	2020-004695-18
**Study design**	This study is a 4-arm, multicenter, randomized, partly double-blind, controlled trial to evaluate the safety and efficacy of convalescent serum (CP) or camostat mesylate with control or placebo in adult patients diagnosed with SARS-CoV-2 and high risk for moderate/severe COVID-19
**Study phase**	Phase 2
**Medical Condition**	In December 2019, the Wuhan Municipal Health Committee identified an outbreak of viral pneumonia cases of unknown cause. Coronavirus RNA was quickly identified in some of these patients. This novel coronavirus has been designated SARS CoV-2, and the disease caused by this virus has been designated COVID-19. Currently, remdesivir is the only approved drug for hospitalized patients with COVID-19 pneumonia requiring oxygen. In contrast no treatment is available for early disease stages and non-hospitalized patients to date. This trial focusses on SARS-CoV-2 positive patients with pre-existing risk factors for a moderate or severe COVID-19 disease course.
**Sponsor**	Heinrich-Heine-University Düsseldorf<br>Universitätsstraße 1, D-40225 Düsseldorf<br><br>represented by the Principal Coordinating Investigator of the study
**Principal Coordinating Investigators PCI**	Verena Keitel-Anselmino, Prof. Dr. med.<br>Klinik für Gastroenterologie, Hepatologie und Infektiologie<br>Universitätsklinikum Düsseldorf<br>Moorenstr. 5, 40225 Düsseldorf<br>0211-81-16330; Fax 0211-81-17517<br>keitelan@uni-duesseldorf.de<br><br>Torsten Feldt, PD Dr. med.<br>Klinik für Gastroenterologie, Hepatologie und Infektiologie<br>Universitätsklinikum Düsseldorf<br>Moorenstr. 5, 40225 Düsseldorf<br>0211-81-16330; Fax 0211-81-17517<br>feldt@hhu.de<br><br>Björn-Erik Ole Jensen, Dr. med.

	Klinik für Gastroenterologie, Hepatologie und Infektiologie<br>Universitätsklinikum Düsseldorf<br>Moorenstr. 5, 40225 Düsseldorf<br>0211-81-16330; Fax 0211-81-17517<br>Jensen@hhu.de<br><br>Steering Committee:<br><br>Edwin Bölke, Prof. Dr. med.<br>Johannes Bode, Prof. Dr. med.<br>Tom Lüdde, Prof. Dr. med.<br><br>RES-Q-HR Working group:<br><br>Wilfried Budach, Prof. Dr. med.<br>Johannes Fischer, Dr. med.<br>Henrike Kolbe<br>Jörg Timm, Prof. Dr. med.<br>Christina Westhoff, Dr.<br>Detlef Kindgen-Milles, Prof. Dr. med.<br>Christiane Matuschek, PD Dr. med.<br>Christian Plettenberg, Dr. med.<br>Kathrin Scheckenbach, PD Dr. med.<br>Andrea Icks, Prof. Dr. Dr.<br>Stephanie Laer, Prof. Dr. med.
**Hypotheses**	The working hypothesis to be tested in the RES-Q HR study is that the early use of convalescent plasma (CP) or camostat mesylate reduces the likelihood of disease progression to modified WHO stages 4b-8 in SARS-CoV-2 positive adult patients at high risk of moderate or severe COVID-19 progression.<br><br>The opposing null hypothesis is rejected if significantly fewer patients in one or both intervention arms show progression of COVID-19 disease to the modified WHO stages 4b-8 within 28 days (see below).
**Rationale**	The ongoing pandemic of the novel coronavirus (SARS-CoV-2) poses a threat to public health and healthcare systems worldwide. SARS-CoV-2 is highly infectious and can cause severe acute respiratory distress syndrome in affected individuals. While remdesivir has recently been approved for COVID-19 associated pneumonia requiring oxygen supplementation, there are currently no therapies available to prevent progression to severe COVID-19 in early infection. Camostat mesylate acts as an inhibitor of the host cell serine protease TMPRSS2 and prevents the virus from entering the cell. Convalescent plasma (CP) represents another antiviral strategy in terms of passive immunization. The hypothesis to be tested in the RES-Q-HR

	study is whether CP or camostat mesylate reduce the probability of disease progression to WHO stages 4b-8 in SARS-CoV-2 positive, adult patients at high risk of severe COVID-19. In the first intervention arm CP (2 units at d1, in individuals ≥150 kg one additional unit on d3) is administered intravenously, while the corresponding control group receives standard of care (SoC) therapy. In the second intervention arm, camostat mesylate (200 mg p.o. 1-1-1) is taken for 7d. The associated control group receives placebo in a double-blinded fashion as well as SoC. The treatment arms are randomized 2:1 to the control arms. A total of 994 participants will be included in at least 10 German centers. The primary endpoint of the study is the number of individuals reaching stage 4b of the modified WHO ordinal scale by day 28 (hospitalization with COVID-19 pneumonia and additional oxygen demand).
**Primary objective / endpoint**	Primary objective<br><br>The working hypothesis to be tested in the RES-Q HR study is that the early use of convalescent plasma (CP) or camostat mesylate reduces the likelihood of disease progression to modified WHO stages 4b-8 in SARS-CoV-2 positive adult patients at high risk of moderate or severe COVID-19 progression.<br><br>Primary endpoint<br><br>The primary endpoint of the study is the cumulative number of individuals who progressed to or beyond category 4b on the modified WHO COVID-19 ordinal scale within 28 days after randomization.<br><br>Clinical status: modified 8-point WHO COVID-19 ordinal scale:<br>0 No SARS-CoV-2 infection<br>1 Outpatient, no restrictions on activities<br>2 Outpatient, restriction of activities<br>3 Hospitalization, no additional oxygen required<br>4a Hospitalization with additional oxygen demand via nasal cannula or mask<br>4b Hospitalization with COVID-19 **pneumonia** and additional oxygen demand via nasal cannula or mask<br>5 Hospitalization, non-invasive ventilation or high flow oxygen<br>6 Hospitalization, invasive mechanical ventilation<br>7 Hospitalization, invasive mechanical ventilation and organ support (catecholamine administration, renal replacement and/or ECMO)<br>8 Death
**Secondary objectives**	Secondary study endpoints

	• Cumulative number of persons in the respective treatment arms versus SoC/placebo in WHO categories 4b-8 by day 8, day 14, day 56 and day 90<br>• Cumulative number of persons in the respective treatment arms versus SoC/placebo in WHO categories 3-4a by day 8, day 14, day 28, day 56 and day 90<br>• *"Event-free"* survival at day 90 and evaluation of all-cause mortality at day 90 using Kaplan-Meier<br>• The proportion of patients with remdesivir therapy and WHO status at initiation of remdesivir<br>• The proportion of patients on dexamethasone therapy and WHO status at baseline dexamethasone<br>• Time to resolution of COVID-19 related symptoms (e.g. fever)<br>• Time to first negative SARS-CoV-2-PCR<br>• Duration of oxygen therapy (in days)<br>• Frequency of occurrence of COVID-19 pneumonia<br>• Percentage of participants in each group with need for mechanical ventilation (and ventilation days)<br>• Duration of hospital stay (in days), duration in intensive care/IMC (in days)<br>• All-cause mortality at day 28<br>• Cumulative incidence of SAEs per group within 90 days follow up<br>• Cumulative incidence of grade 3/4 AEs per group<br>• SARS-CoV-2 antibody concentrations (IgA, IgG, NT) in serum on day 8, day 14, day 90<br>• Number of screening failures due to the lack of a suitable plasma preparation<br><br><u>Exploratory endpoints (optional)</u><br><br>• Plasma concentrations of camostat mesylate<br>• Total immunoglobulin titer in the serum of all study participants<br>• Baseline levels and change from baseline for inflammation/immune related, ARDS associated, coagulation related biomarkers including chemokine, cytokine, ACE and ACE cleavage products, miRNAs, SuPAR, etc. in serum / plasma<br>• Baseline and changes from baseline in cellular immunophenotypes (whole blood analyses, PAXgene® tubes, RNASeq, etc)<br>• Baseline and change from baseline urine biomarkers<br>• Analysis of host microbiome (from NP swabs)<br>• Emergence of viral resistance

	• Presence of co-infections<br>• Host genomics
**Inclusion criteria**	1. Individuals (female, male, diverse) ≥ 18 years with SARS-CoV-2 infection, confirmed by PCR before study enrollment<br>2. SARS-CoV-2 positive PCR ≤ 3 days old (date of NP swab)<br>3. Presence of ≥ 1 SARS-CoV-2 typical symptom (fever, cough, shortness of breath, sore throat, headache, fatigue, smell/and or taste disorder, diarrhea, abdominal symptoms, exanthema) and symptom duration ≤ 3 days.<br>4. Ability to provide written informed consent<br>5. Presence of **at least one** of the following criteria:<br>- Patients > 75 years<br>- Patients > 65 years with at least one other risk factor (BMI >35 kg/m^2, coronary artery disease, CKD with GFR <60 ml/min but ≥ 30 ml/min, diabetes mellitus, active tumor disease)<br>- Patients with a BMI >35 kg/m^2 with at least one other risk factor (CAD, CKD with GFR <60 ml/min but ≥ 30 ml/min, diabetes mellitus, active tumor disease)<br>- Patients with a BMI >40 kg/m^2<br>- Patients with chronic obstructive pulmonary disease (COPD) and/or pulmonary fibrosis
**Exclusion criteria**	1. Age <18 years<br>2. Unable to give informed consent<br>3. Pregnant women or breast-feeding mothers<br>4. Previous transfusion reaction or other contraindication to a plasma transfusion<br>5. Known hypersensitivity to camostat mesylate and/or severe pancreatitis<br>6. Volume stress due to CP administration would be intolerable<br>7. Known IgA deficiency<br>8. Life expectancy < 6 months<br>9. Duration SARS-CoV-2 typical symptoms > 3 days<br>10. SARS-CoV-2 PCR detection older than 3 days

	11. SARS-CoV-2 associated clinical condition ≥ WHO stage 3 (patients hospitalized for other reasons than COVID-19 may be included if they fulfill all inclusion and none of the exclusion criteria).<br>12. Previously or currently hospitalized due to SARS-CoV-2<br>13. Previous antiviral therapy for SARS-CoV-2<br>14. ALT or AST > 5 x ULN at screening<br>15. Liver cirrhosis > Child A (patients with Child B/C cirrhosis are excluded from the trial)<br>16. Chronic kidney disease with GFR < 30 ml/min<br>17. Concurrent or planned anticancer treatment during trial period<br>18. Accommodation in an institution due to legal orders (§40(4) AMG).<br>19. Any psycho-social condition hampering compliance with the study protocol.<br>20. Evidence of current drug or alcohol abuse.<br>21. Use of other investigational treatment within 5 half-lives of enrollment is prohibited<br>22. Previous use of convalescent plasma for COVID-19<br>23. Concomitant proven influenza A infection<br>24. Patients with organ or bone marrow transplant in the three months prior to Screening Visit
**Study medication**	Participants will be randomized to receive either therapy of convalescent plasma (CP, 2 units d1, ≥ 150kg a third unit on d3), camostat mesylate (200 mg p.o. 1-1-1 on days 1-7), standard of care (SOC, control for CP) or placebo (p.o. 1-1-1, control for camostat mesylate on day 1-7)<br><br>Subjects will be randomized to the different treatment arms.
**Placebo/ Reference Medication**	Placebo for camostat mesylate arm (p.o. 1-1-1) d1-d7 and SoC as control for CP.
**Efficacy Assessment**	The WHO ordinal scale is an assessment of the clinical status at the first assessment of a given study day. Each visit, the worst score for the previous period will be recorded. The scale is as follows:<br><br>Clinical status on the modified 8-point WHO COVID-19 ordinal scale:<br>0 No SARS-CoV-2 infection<br>1 Outpatient, no restrictions on activities<br>2 Outpatient, restriction of activities<br>3 Hospitalization, no additional oxygen required

	4a Hospitalization with additional oxygen demand via nasal cannula or mask<br>4b Hospital stay with COVID-19 **pneumonia** and additional oxygen demand via nasal cannula or mask<br>5 Hospitalization, non-invasive ventilation or high flow oxygen<br>6 Hospitalization, invasive mechanical ventilation<br>7 Hospitalization, invasive mechanical ventilation and organ support (catecholamine administration, renal replacement and/or ECMO)<br>8 Death
**Safety Assessment**	Camostat mesylate plasma levels on day 3 and day 8 (45 min after dosing on day 3, after the end of treatment on day 8)<br>For this study, all AEs and all SAEs occurring from the time the informed consent is signed through the Day 90 (end of study) visit will be documented, recorded, and reported.
**Safety and Discontinuation Criteria**	This study may be prematurely terminated for the patient or in total if there is sufficient reasonable cause.
**Trial Duration**	The study will last for up to 18 months.
**Duration of Intervention per Patient**	An individual subject will complete the interventional part of the study within 8 days.<br>An individual subject will complete the entire study in about 90 days, from screening at day 0 to follow-up on day 90 ±5 days.
**Follow- up per Patient**	Day 14<br>Day 28 ± 5 days (telephone)<br>Day 56 ± 5 days (telephone)<br>Day 90 ± 5 days
**Total number of patients**	*to be assessed for eligibility (n = 1094)*<br>*To be allocated to trial (n = 994)*<br>*To be analyzed (n = 994 in ITT analysis)*
**Statistical analysis**	Description of the primary efficacy analysis and population:<br>Efficacy will be analyzed in the ITT (Intention-to-Treat) population. For the final analysis of the primary endpoint, we plan to apply two-sample Z-test for proportions with a 5% two-sided significance level. We will report the 95% confidence interval of the OR and rates differences δ between treatment and control groups. In addition, a Bayesian interpretation of the trial results will be performed by using an objective Bayesian approach. If the number of missing data on the primary end

	point is large than 5%, a full Bayesian analysis will be applied. Robustness of the trial conclusion will be assessed under two possible missing data mechanisms: MAR (Missing at Random) and NMAR (Not Missing at Random).<br><br><u>Group sequential analysis:</u><br><br>We plan an interim analysis for efficacy when 50% of the participants have been randomized. We use a two-sided O'Brien-Fleming spending function at a statistical significant level of 5%.<br><br><u>Safety:</u><br><br>Safety will be reported using listings and descriptive statistical methods<br><br><u>Secondary endpoints:</u><br><br>Statistical analysis of the secondary endpoints will be performed using a similar strategy as the primary endpoint. Descriptive statistics will be calculated for each treatment group and appropriate statistical techniques will be applied.
**Participating Organization**	Koordinierungszentrum für Klinische Studien (KKS)<br>Universitätsklinikum Düsseldorf<br>Moorenstr. 5<br>D-40225 Düsseldorf<br><br>Apotheke des Universitätsklinikums Düsseldorf<br>Moorenstr. 5<br>D-40225 Düsseldorf<br><br>Institut für Transplantationsdiagnostik und Zelltherapeutika (ITZ)<br>Moorenstr. 5<br>D-40225 Düsseldorf<br><br>Klinik für Hals-Nasen-Ohrenheilkunde<br>Moorenstr. 5<br>D-40225 Düsseldorf<br><br>Institut für Virologie<br>Universitätsklinikum Düsseldorf<br>Universitätsstr. 1<br>D-40225 Düsseldorf<br><br>Institut für Versorgungsforschung und Gesundheitsökonomie<br>Centre for Health and Society

	Medizinische Fakultät<br>Heinrich Heine University<br>Moorenstr. 5<br>D-40225 Düsseldorf<br><br>Institute of Clinical Pharmacy and Pharmacotherapy<br>Heinrich Heine University<br>Universitätsstrasse 1<br>D-40225 Düsseldorf
**Funding**	This study will be funded by Bundesministerium für Gesundheit (BMG)

1 INTRODUCTION

1.1 Background and Purpose of the Study

The ongoing pandemic with the novel coronavirus SARS-CoV-2 poses a massive threat to public health and has a major impact on the economic situation worldwide. Infection with SARS-CoV-2 is highly contagious and can cause severe respiratory distress syndrome (1, 2). In addition, the viral infection triggers complex immune and inflammatory responses that can cause severe damage to a wide variety of organ systems. On October 12th, a total of 325,331 laboratory-confirmed COVID-19 cases were reported in Germany (RKI), including 9,621 deaths.

Since the beginning of the pandemic, various risk factors have been identified that increase the probability of a severe course and death from COVID-19. While the *infection fatality rate* (*IFR*), which apart from special situations such as an outbreak on a cruise ship (3), can usually only be approximated using models, the WHO estimates the IFR at 0.6% across all age groups. Recent studies correlating seroprevalence and confirmed COVID-19 deaths as well as excess deaths from Spain and United Kingdom estimate the IFR around 1% in populations with similar age structure to Germany (4, 5). This figure is significantly higher depending on the population studied and reaches more than 10% for people over 70 years of age (6). The *case fatality rate* (*CFR*), which only includes detected cases and depends strongly on how many infected persons are detected in the respective target area as part of the test strategy, must be distinguished from this. Depending on the risk structure of the population, local testing strategies and the burden on the local health care system during the pandemic, the CFR varied between 2.6% during an outbreak on a cruise ship and 12% during the first months of the outbreak in Italy (7). For Germany, the RKI currently estimates the proportion of casualties at 4.4%. Recent publications show that the mortality rate of patients admitted to hospital due to COVID-19 in Germany is about 22% (8). The inpatient mortality of patients on mechanical ventilation ranged from 28% for patients aged 18-59 years to 72% for patients over 80 years (8). A significantly increased risk of death from COVID-19 is also reported in patients with liver cirrhosis. In published cohort data, the odds ratio (9 for child B cirrhosis and 28 for child C cirrhosis (9). In a prospective cohort from the USA, the OR for hospitalization due to COVID-

19 reached 37.9 for patients aged over 75 years, while other relevant risk factors for hospitalization were the presence of chronic kidney disease (CKD; OR 2.6) and an increased body mass index (BMI; OR 2.5 for BMI>40kg/m^2) (10). A large prospective cohort from the UK, found an OR for COVID-19 associated death of 8.5 for people over 70 years of age and of 11.1 for people over 80 years of age (11). Reported OR for intensive care unit (ICU) admission range from 17.8 in the presence of chronic obstructive pulmonary disease (COPD), to 4.4 in the presence of coronary artery disease (CAD) (12), to 2.8 for diabetics (13) and to 3.1 for patients with active malignancy (14).

Currently, no vaccines or prophylactic therapies are available that could protect these high-risk groups from COVID-19. While remdesivir has recently been approved for COVID-19 associated pneumonia, there are currently no therapies available to prevent progression of early SARS-CoV-2 infection to moderate or severe COVID-19 (15, 16). Furthermore, no therapy has been approved for patients with SARS-CoV-2 in an outpatient setting. Therefore, there is an urgent need for clinical studies to evaluate antiviral strategies that can prevent disease progression or reduce disease severity in individuals tested positive for SARS-CoV-2.

Since 2003, there were two other epidemics with coronaviruses that were associated with high mortality, SARS-CoV-1 in 2003 and Middle East Respiratory Syndrome (MERS) in 2012. In both outbreaks, the high mortality and absence of effective therapies led to the use of convalescent plasma (CP) (17). The largest study involved the treatment of 80 patients with SARS-CoV-1 in Hong Kong (18). Patients treated before day 14 had improved prognosis defined by discharge from hospital before day 22, consistent with the hypothesis that earlier administration is more likely to be effective (18). In addition, those who were PCR positive and seronegative for coronavirus at the time of therapy had improved prognosis. The case fatality rate was 12% (n=80) with CP, while the overall mortality between March and May 2003 in Hong Kong without CP was 17% (n=1755) (18).

Results from the COVID-19 early access programme in the USA demonstrated a significantly lower 7-day and 28-day mortality if CP was administered within 3 days of SARS-CoV-2 diagnosis in comparison to transfusion after ≥4 days of COVID-19 diagnosis (19). A randomized, open-label trial using CP for severe to life-threatening COVID-19 demonstrated shortening of the time to clinical improvement in patients with severe disease by about 5 days (20).

Camostat mesylate acts as inhibitor of the host cell serine protease TMPRSS2, which is needed to prime the viral S protein for cell entry (21). Therefore, this protease inhibitor may prevent viral entry and thus viral spread in the respiratory tract early after infection.

Previous studies on the benefit of antiviral therapy in lytic infections, such as the effect of neuraminidase inhibitors on influenza infection, suggest that antiviral strategies such as camostat mesylate or CP should be used early in the course of the disease (22). This clinical trial is designed to evaluate the efficacy and safety of two antiviral therapeutics strategies (CP and camostat mesylate) early after infection with SARS-CoV-2 in adult patients with high risk to develop a moderate or severe form of COVID-19 infection

1.2 Study Design

This study is a 4-arm, multicenter, randomized, partly double-blind, controlled trial to evaluate the safety and efficacy of convalescent serum (CP) or camostat mesylate with Standard of Care

(SoC) or placebo in adult patients newly diagnosed with SARS-CoV-2 and high risk for moderate/severe COVID-19.

Patients eligible for the trial need to fulfil 4 criteria:

- date of their positive SARS-CoV-2 PCR results is not older than **3 days (≤ 3 days)**
- presence of SARS-CoV-2 associated symptoms (as defined in the inclusion criteria (see below)), but symptom duration ≤ **3 days**
- patients are **at high risk** for development of moderate/severe COVID-19 disease as defined in the inclusion criteria
- SARS-CoV-2 associated clinical condition ≤ WHO stage 2

After the participant has provided informed consent, baseline characteristics, past medical history, current clinical history and concomitant medication will be collected and documented. Physical examination, vital signs with temperature, respiratory rate, SpO_2 on room air will be taken and documented. Women of childbearing age will have a urine pregnancy test.

After the screening procedures, eligible individuals will be randomized in a 2:2:1:1 ratio to receive either CP, camostat mesylate, SoC or placebo + SoC.

Randomization will be per center. Individuals randomized to CP will receive 2 units of CP on day 1 (d1). Individuals ≥150 kg, will receive a 3rd unit of CP. To avoid volume overload and unwanted side effects this will take place on day 3 (d3). Individuals randomized to camostat mesylate, will receive camostat mesylate 200 mg p.o. (1-1-1) on days 1-7.

On d1, d3, d5, d8, d14, d90 vital signs, respiratory status, symptom status, clinical status (WHO ordinal scale), potential adverse events (AEs) and concomitant medication will be documented. Laboratory tests for safety, nasopharyngeal (NP) swabs and optionally also blood/urine for exploratory endpoints will be taken at screening (d0) and on d3, d5, d8, d14, d90. Women of childbearing age will have an additional urine pregnancy test on d8. At screening, d28 and d29 quality of life will be measured by SF-12 and a visual analogue scale (VAS).

On day 28 and day 56 a telephone visit will be carried out to assess concomitant medications, respiratory status, clinical status and adverse events.

Plasma levels of camostat mesylate will be determined on d3 and d8 for safety assessment and to perform exposure-response analyses. Anti-SARS-CoV-2 antibody titers will be measured in all patients to monitor immunological response.

An independent data and safety monitoring board (DSMB) will actively monitor interim data to make recommendations to the sponsor about early study closure or changes to study arms.

1.3 Study Scheme

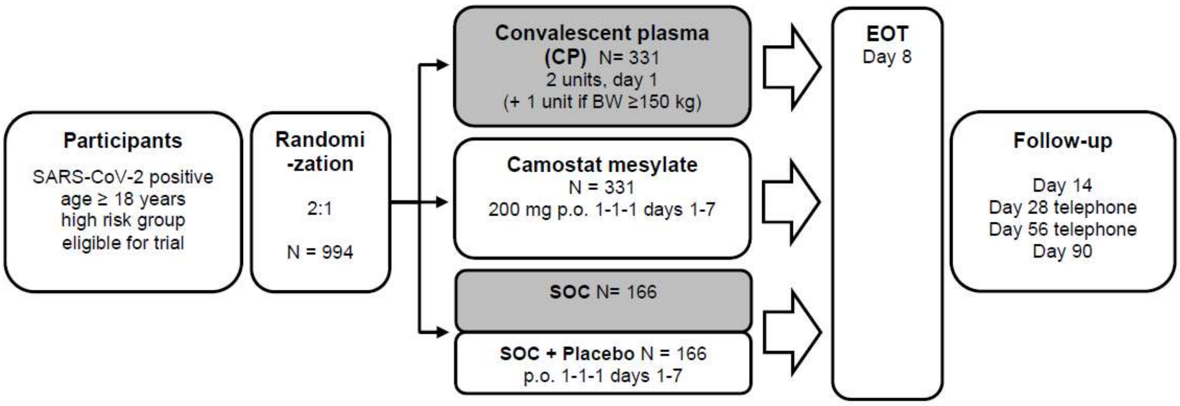

Figure 1 RES-Q HR Flow Chart

1.4 Planned Intervention and Selection of Study Population

The working hypothesis to be tested in the RES-Q HR study is that the early use of convalescent plasma (CP) or camostat mesylate reduces the likelihood of disease progression to modified WHO stages 4b-8 in SARS-CoV-2 positive, adult patients at high risk for moderate/severe COVID-19.

The opposing null hypothesis is rejected if significantly fewer patients in one or both intervention arms show progression of COVID-19 disease to the modified WHO stages 4b-8 within 28 days (see below).

The anticipated reduction in disease progression and thus hospitalization, admission to ICU and need for mechanical ventilation would significantly benefit patients and reduce the massive burden on the healthcare system.

State of the art on the planned interventions:

Both CP and camostat mesylate are antiviral strategies. CP has been used to successfully confer immunity against a number of infectious diseases. In the context of COVID-19, CP is collected from donors who have survived COVID-19 and developed neutralizing antibodies against SARS-CoV-2. The processed plasma can be transfused into patients infected with SARS-CoV-2. Based on the previous experience with CP in individuals infected with SARS-CoV-1, MERS and influenza as well as in early trials and case reports from COVID-19, CP may prevent disease progression towards moderate/severe COVID-19 especially when given at an early timepoint after infection (23-27).

Camostat mesylate inhibits the protease activity of TMPRSS2, which is important for virus entry into the host cell. The angiotensin-converting enzyme 2 (ACE 2) serves as a receptor for SARS-CoV-2 (21, 28, 29). Before binding to ACE2, the S protein of the virus must be modified by the serine protease TMPRSS2 on host cells (21). The inhibition of the protease activity of TMPRSS2 by camostat mesylate efficiently prevented the entry of SARS-CoV-2 into primary lung cells and lung cell line Calu-3 in *vitro* without relevant side effects ($EC_{50} \approx 1 \mu M$) (21). Camostat mesylate is approved in Japan for the treatment of chronic pancreatitis at a dose of 600 mg daily (clinical information Foipan®). The intake of 200 mg camostat mesylate (clinical

information Foipan®) resulted in a maximum plasma concentration of 87.1 ng/ml (≈ 0.176 µM) in healthy volunteers 40 minutes after administration (clinical information Foipan®). This plasma concentration is about 5 times lower than the EC_{50} required to block viral entry into Calu-3 cells (21). However, it has never been verified which dosages reach inflamed lung tissue and it is assumed that the effective levels are significantly higher here. Furthermore, it has not been determined whether plasma concentrations increase after continuous treatment over several days. Therefore, plasma levels will be determined in a subgroup of patients in the camostat mesylate arm (d3 and d8).

Convalescent plasma is used as passive transfer of neutralizing antibodies to confer immunity against SARS-CoV-2. Use of CP in over 20,000 individuals as part of an early access program in the United States of America was safe (30).

Thus, both CP and camostat mesylate, when used early in SARS-CoV-2 positive individuals, could prevent disease progression by reducing viral load and viral entry, respectively. This will be investigated in the proposed study in high-risk patient populations.

Selection of the study population:

Previous analyses of SARS-CoV-2 positive individuals suggest that in various European countries 17-69% of those infected have a moderate/severe course of the disease and must be hospitalized (8). The mortality rate of hospitalized SARS-CoV-2 positive patients in Germany was 22% (8). While the risk of a severe COVID-19 infection for the whole population is rather low, there are, as mentioned above, individuals with a significantly increased risk of a severe course of the disease or with a disproportionately high mortality. These includes individuals aged 65 or higher (especially high risk in those > 80 years of age) as well as individuals with pre-existing conditions such as COPD, coronary artery disease (CAD), diabetes mellitus, chronic kidney disease (CKD), as well as obesity (BMI >35 kg/m^2). As these individuals are at high risk of moderate to severe disease progression, they would benefit from an early antiviral therapy strategy (see below for details).

2 RISK/BENEFIT ASSESSMENT FOR THE STUDY

2.1 Known Potential Risks

The safety profile was given special attention in the selection of the study medications since the majority of patients will be managed as outpatients. The development of a treatment option, which can be administered with minimal use of resources, would be important. Both study interventions are generally well established and comprehensive experience exists, although not for the use in COVID-19. Both medications have no limitations in the administration in elderly patients and patients with common comorbidities, in particular liver and renal disease. A close clinical and biochemical monitoring shall assure the safety of participants and allow an early identification of potential side effects.

In general, the following potential risks for participants need to be considered:

1. Potential side effects of the study medication
2. Potential risks of having blood drawn
3. Potential risks of having nasopharyngeal swabs taken
4. Risks to privacy (see below)

2.2 Potential Side Effects of the Study Medication

In order to detect possible side effects of the study medication and to avoid complications, patients enrolled in the RES-Q HR trial will undergo close clinical (days 1,3,5,8,14,90) and laboratory (days 3,5,8,14) assessments during the period of drug administration (d1-d7) and thereafter to monitor hepatic (AST, ALT, GGT, ALP, bilirubin, INR), renal (creatinine, urea) and myocardial (CK, NT-proBNP) dysfunction. Laboratory abnormalities or possibly related adverse events (AEs) will be treated appropriately and followed to resolution. On day one (first administration of study medication), patients are monitored for at least 1 hour after ingestion / infusion of the study medication. Particular risks associated to each study medication are described below.

2.2.1 Camostat Mesylate

Camostat mesylate is a serine protease inhibitor, in particular of the transmembrane protease TMPRSS2. The drug is approved for the treatment of chronic pancreatitis and postoperative reflux esophagitis in Japan under the trade name Foipan® and manufactured by Ono Pharmaceutical. In this study, generic drugs are used in addition to the original drug. The usual dosage for oral use is 600 mg of camostat mesylate daily in three doses of 200 mg each for the treatment of chronic pancreatitis.

<u>Contraindications and precautions:</u>

Camostat mesylate is contraindicated in patients with a history of hypersensitivity to any of the ingredients of the product. It should **<u>not</u>** be administered in patients with severe chronic pancreatitis requiring suction of gastric juice, or dietary restrictions such as fasting and abstention from drinking, for the treatment of postoperative reflux esophagitis due to reflux of gastric juice since the efficacy of this product cannot be expected. It should be administered with care in patients with known hypersensitivity.

<u>Adverse reactions:</u>

The drug is generally well tolerated and has a favourable safety profile. Adverse reactions, including abnormal laboratory test values, were observed in 69 (1.8%) of 3.806 patients evaluated in the investigation for the use for the indication “Remission of acute symptoms of chronic pancreatitis” conducted up to the time of approval and in the Drug Use Investigation. The major adverse reactions were rash in 15 incidences (0.4%), pruritus in 9 incidences (0.2%), nausea in 10 incidences (0.3%), abdominal discomfort in 7 incidences (0.2%), and abdominal fullness in 6 incidences (0.2%) at the end of the re-examination period. Other reported adverse reactions included leukopenia and erythrocytopenia (≤0.1%), eosinophilia (incidence unknown), rash, pruritus and other signs of hypersensitivity (≥0.1%-0.5%), nausea, abdominal discomfort, abdominal fullness, diarrhoea (≥0.1%-0.5%), anorexia, vomiting, dry mouth, heartburn, abdominal pain, constipation (≤0.1%), increased AST, ALT, and other liver enzymes (≥0.1%-0.5%), increased BUN and/or creatinine (≤0.1%), edema and hypoglycaemia (≤0.1%).

<u>Clinically significant adverse reactions:</u>

Shock or anaphylactoid symptoms: Shock or anaphylactoid symptoms (both incidences unknown) may occur and have been reported in post-marketing use. In most cases, the

adverse reactions occurred within 30 min after taking the product. Therefore, individuals will be monitored for 60 min after ingestion of the first dose of camostat mesylate.

Thrombocytopenia: Thrombocytopenia (incidence unknown) may occur rarely.

Hepatic function disorder or jaundice: Hepatic function disorder accompanied by remarkable increase of AST(GOT), ALT(GPT), GGT, ALP, or jaundice (both incidences unknown) may occur.

Hyperkalaemia: Severe hyperkalaemia (incidence unknown) may occur. Patients should be carefully monitored by conducting serum electrolyte tests.

To prevent complications due to adverse reactions, several precautionary measures are implemented. Patients are clinically observed and monitored for the development of hyperreactivity reactions with possible anaphylactic symptoms or shock for 1 hour after taking the first dose of camostat mesylate, and appropriately treated in case signs of hypersensitivity occur. Patients are also carefully monitored with biochemical safety controls (d3, d5, d8, d14). If any abnormalities are observed, appropriate therapeutic measures such as discontinuing the administration will be taken and abnormalities or possibly related AEs will be followed to resolution.

2.2.2 Convalescent Plasma

Current data on use of convalescent plasma suggest it is safe in general and also in SARS-CoV-2 infection as recently demonstrated in 20,000 individuals (30). Transfusion reactions were observed in <1% (n=78), thromboembolic or thrombotic events were diagnosed in <1% (n=113), and cardiac events were reported in about 3% (n=677) of these individuals. However, 66% of the thromboembolic or thrombotic events (n=75) and 88% of the cardiac events (n=597) were judged to be unrelated to the CP transfusion per se (30).

Since COVID-19 primarily affects the lung, transfusion-related acute lung injury (TRALI) was of special interest for the risk/benefit ratio and was observed in 0,1% of COVID-19 patients receiving convalescent plasma (30).

The theoretical risks of CP in COVID-19 patients includes the phenomenon of antibody-mediated enhancement of infection (ADE) (31). ADE can occur in several viral diseases and involves more severe disease in the presence of certain antibodies. For coronaviruses, several mechanisms for ADE have been described and there is the theoretical concern that antibodies to one type of coronavirus could enhance infection to another viral strain (32). Data from larger RCTs for ADE in SARS-CoV-2 is still lacking and caution and vigilance for any evidence of enhanced infection will be required. Therefore, patients within the trial will be closely monitored (clinical visits, d3, d5, d8, d14) and followed up until day 90 after transfusion.

Another theoretical risk is that antibody administration to those exposed to SARS-CoV-2 may prevent disease but modify the immune response that those individuals mount attenuated immune responses, which would leave them vulnerable to subsequent re-infection. In this regard, passive antibody administration before vaccination with respiratory syncytial virus was reported to attenuate humoral but not cellular immunity. The half-life of IgG is 25.8 days (33), therefore, vaccination should be effective 8-12 weeks after CP administration. This concern will be investigated as part of this clinical trial by measuring immune responses in those exposed and treated with convalescent plasma to prevent disease.

Passive antibodies are derived from human plasma. The plasma obtained from convalescent patients will be subjected to testing protocols that are equivalent to those used by blood banks and transfusion services. However, there is a very small risk of allergy/anaphylaxis, transfusion related acute lung injury (TRALI), transfusion associated circulatory overload (TACO), passive transfer of potential unknown infectious agents or infections, or accidental receipt of an incompatible blood product, comparable with the risk of fresh frozen plasma (FFP) infusion. According to the German "*Hämovigilanz-Bericht*" in 26 out of 10^6 plasma transfusions a severe transfusion reaction occurs. Most adverse effects are mild and transient including headaches, flushing, fever, chills, fatigue, nausea, diarrhea, blood pressure changes and tachycardia. Late adverse events are rare and include acute renal failure and thromboembolic events.

In order to prevent the described risks as much as possible, patients in whom volume stress due to CP administration would be considered an unacceptable risk (e. g. cardiac failure) are excluded from the study. Safety laboratory tests (ABO typing, pregnancy testing, CBC) will be performed. Patients are clinically observed and monitored for the development of hyperreactivity reactions or signs of volume overload for at least 1 hour after receiving the CP. If any abnormalities are observed, appropriate therapeutic measures such as discontinuing the administration will be taken and abnormalities or possibly related AEs will be followed to resolution.

According to the most recent hemovigilance report published by the German Federal Authority Paul-Ehrlich Institute (PEI) the residual risk of adverse reactions in the observation period from 2000 to 2017 is:

Serious allergic transfusion reactions per 10^6 transfused FFPs:	8.98
TRALI cases per 10^6 transfused FFPs:	4.85*
Viral transmissions (HBV, HCV, HIV, HEV) per 10^6 transfused FFPs:	1.00
TACO cases per 10^6 transfused FFPs:	1.66**
Transfusion related bacterial infections per 10^6 transfused FFPs:	0.05

*In the period after 2011, i.e. after restriction of plasma for clinical use to male donors, female donors without previous pregnancies or female donors who tested negative for HLA- and HNA-antibodies, the residual TRALI risk after plasma transfusion was reduced to 1.45 per 10^6 FFPs (years 2012-2015) and 1.32 per 10^6 FFP (years 2016 to 2017).

** TACO was not monitored for the whole period.

The only conceivable adverse event which is not very rare may be an allergic reaction to FFPs. Patient with a history of allergic reactions to FFPs or a condition which can predispose to an allergic reaction (IgA deficiency) are excluded from this trial.

Overall, the ratio between risk of this treatment and potential benefit seems favorable.

2.3 Potential Risks of Having Blood Drawn

Drawing blood may cause transient discomfort and fainting. Fainting is usually transient and managed by having the subject lie down and elevate his/her legs.

Bruising at the blood collection sites may occur but can be prevented or lessened by applying pressure to the blood draw site for a few minutes after the blood is taken.

Blood sampling will only be performed by well-trained and experienced personnel in order to minimize the risk.

2.4 Potential Risks of Nasopharyngeal Swab

Oropharyngeal and throat swab taking may cause transient local discomfort and retching; relevant complications are not expected. Rarely, nose bleeds can be triggered by nasopharyngeal swabs, which in the majority of cases resolves spontaneously. Taking of swabs will only be performed by well-trained and experienced personnel.

2.5 Risks to Privacy

Subjects will be asked to provide personal health information (PHI). All attempts will be made to keep this PHI confidential according to regulatory requirements and national law. However, there is a chance that unauthorized persons will see the subject's PHI. All study records will be kept in a locked file cabinet or maintained in a locked room at the participating clinical site. Electronic files will be password protected.

The patient must be informed that medical records may be examined by authorized monitors or Clinical Quality Assurance auditors, by appropriate IEC members and by inspectors from regulatory authorities. The patient identification list is under strict control of the investigator and will not be transferred to the Sponsor. Data recorded by eCRF have been pseudonymized. In the sponsors' facilities, data security and protection rules are strictly followed with the help of SOPs and working instructions. The Sponsor takes care that no unauthorized access to their computer system takes place. It is also responsible for adequate backup procedures to prevent loss of data. Only specified persons involved in the trial get authorization to enter or access data in the clinical trial data base, based on predefined roles. There will be a complete audit trail of all transactions. Persons in the KKSD involved in the trial are sworn to secrecy.

In case of withdrawal of informed consent, it will be checked, whether stored data are still necessary, according to AMG §40 Abs. 2a. If this is not the case, these data are deleted.

Any publications from this trial will not use information that will identify subjects by name. Organizations that may inspect and/or copy research records maintained at the participating site for quality assurance and data analysis include groups such as the IRB, Sponsor or the pertinent regulatory authorities.

2.6 Known Potential Benefits

There is no data from large RCT demonstrating the positive effects of the study medications in COVID-19. However, evidence from preclinical studies and case series suggest a possible beneficial effect. Thus, camostat mesylate and/or CP may or may not improve clinical outcome of an individual adult subject with moderate COVID-19 who participates in this trial. At the moment, no other treatment option for this group of patients with early COVID-19 is available, and thus no treatment options are withheld when participating in this study. Since novel therapeutic options may arise during the trial, the DSMB and PCI will evaluate potential therapeutic options with regard to the study population in 2-3 months intervals during the trial period.

Participants are closely monitored during the follow-up visits and assessed for deterioration clinically and biochemically by experienced study physicians. In case of disease progression,

hospitalisation in the study centre will be prompted and appropriate treatment will be implemented. It can be assumed, that the medical care is improved compared to standard care outside of studies, since due to the quarantine, consultations are normally kept to a minimum. Patients will have a 24 hours hotline contact for any events in-between the follow-up visits and prompt consultations can be arranged by the study team: **0211-8104071**.

There is also a potential benefit to society from this study resulting from insights gained about the therapeutic agents under study as well as from the natural history of the disease. The identification of an effective therapeutic option for the investigated patient group would have the potential to not only prevent individual complications, but also to decrease the burden of health systems in general and of ICUs in particular.

2.7 Assessment of Potential Risks and Benefits

Although no RCT data on the administration in COVID-19-patients are available, the safety profiles of both IMPs are well known and favourable. Potential risks will be addressed in order to avoid complications. Given that current data, although sparse to date, suggest that the IMPs might be associated with some positive clinical effects and that the benefits in the study population with high risk of developing complications outweigh the risks.

Alternatives:

Since no treatment is available for early SARS-CoV-2 infection, the alternative to participation in this study is routine care and monitoring. Potentially, there will be other treatment studies available for early infection in an outpatient setting, however, currently none of these studies will be available for a large number of patients at each center. In early 2021, vaccination studies are to be expected, however, these will be used as primary prevention and thus target a different cohort of individuals. Emerging therapeutic options will be closely followed by the PCI and DSMB and may lead to halting or discontinuation of the study in case a superior therapeutic agent becomes available for the study population (see below).

3 ENDPOINTS AND STUDY DESIGN

3.1 Endpoints

3.1.1 Primary Study Endpoint

The primary endpoint of the study is the number of individuals whose clinical status is on the COVID-19 modified WHO ordinal scale ≥ 4b up to and including day 28.

Clinical status: Modified 8-point WHO ordinal scale

0 No SARS-CoV-2 infection

1 Outpatient, no restrictions on activities

2 Outpatient, restriction of activities

3 Hospitalization, no additional oxygen required

4a Hospitalization with additional oxygen demand via nasal cannula or mask

4b Hospitalization with COVID-19 pneumonia requiring additional oxygen demand via nasal cannula or mask

5 Hospitalization requiring non-invasive ventilation or high flow

6 Hospitalization requiring invasive mechanical ventilation

7 Hospitalization, invasive mechanical ventilation and organ support (catecholamine administration, renal replacement and/or ECMO)

8 Death

3.1.2 Secondary Study Endpoints

In addition, the following secondary endpoints are analyzed:

- Cumulative number of persons in the respective treatment arms versus SoC/placebo in WHO categories 4b-8 by day 8, day 14, day 56 and day 90
- Cumulative number of persons in the respective treatment arms versus SoC/placebo in WHO categories 3-4a by day 8, day 14, day 28, day 56 and day 90
- *"Event-free"* (not hospitalized, no COVID-19 associated long-term effects such secondary sclerosing cholangitis, pulmonary disease, no reinfection) survival at day 90 and evaluation of all-cause mortality at day 90 using Kaplan-Meier
- The proportion of patients with remdesivir therapy and WHO status at initiation of remdesivir
- The proportion of patients on dexamethasone therapy and WHO status at baseline dexamethasone
- Time to resolution of COVID-19 related symptoms (e.g., fever)
- Time to first negative SARS-CoV-2-PCR
- Duration of oxygen therapy (in days)
- Frequency of occurrence of COVID-19 pneumonia
- Percentage of participants in each group with need for mechanical ventilation (and ventilation days)
- Duration of hospital stay (in days), duration in intensive care/IMC (in days)
- All-cause mortality at day 28
- Cumulative incidence of SAEs per group within 90 days follow up
- Cumulative incidence of grade 3/4 AEs per group
- SARS-CoV-2 antibody concentrations (IgA, IgG, NT) in serum on day 8, day 14, day 90
- Number of screening failures due to the lack of a suitable plasma preparation

3.2 Study Design

3.2.1 Overall Design

This is a phase II, randomised controlled, 4-arm multicentre trial for outpatients with early stage SARS-CoV-2 infection, who are at high risk of disease progression. Camostat mesylate will be compared to placebo in a double-blinded fashion. Convalescent plasma will be compared to standard of care in an open-label fashion.

Study Treatments:

Approximately 1094 individuals meeting all eligibility criteria may be randomized in a 2:2:1:1 ration into either treatment group:

- Treatment group 1: Two units of CP (about 250 ml each) on day 0 or day 1, for individuals ≥ 150 kg a third unit of CP will be administered on day 3
- Treatment group 2: Camostat mesylate 200 mg capsules p.o. 1-1-1 from for 7 consecutive days (d0-d7 or d1-d8)
- Treatment group 3: Standard of Care (SoC)
- Treatment group 4: Placebo 200 mg capsules p.o. 1-1-1 from for 7 consecutive days (d0-d7 or d1-d8)

Duration of Treatment:

Patients in treatment group 1 will receive the CP on one day, patients in groups 3 and 4 will receive camostat mesylate or placebo over a 7-day course. The study duration for all individuals is 90 days ± 5 days.

End of Study:

The study will end when the last participant reached the last visit on day 90 (± 5 days).

Poststudy Care:

The long-term care of all participating individuals will remain in the responsibility of their primary care physician. The study medication will not be supplied beyond the treatment duration as outlined above.

3.2.2 Justification for Dose

Camostat mesylate has been shown to block TMPRSS2 activity and has been approved in Japan for chronic pancreatitis in a dose of 600 mg daily (clinical information Foipan®). The application of 200 mg camostat mesylate to healthy volunteers led to a peak plasma level 40 minutes after administration of 87.1 ng/ml (≈0.176 µM) (clinical information Foipan®). Although this plasma concentration is lower than the EC_{50} reported to block viral entry into Calu-3 cells *in vitro*, we chose to adhere to the approved dose on the one hand because of the high safety requirements in outpatient administration without continuous clinical monitoring, and on the other hand the administration in early SARS-CoV-2 infection.

Dose calculation for CP is based on 2 units (250-325 ml per unit) of plasma with anti-SARS-CoV-2 neutralizing antibody titers of ≥ 1:160 (measurements: Institut für Virologie, Düsseldorf). For the purposes of the proposed trial, titers of ≥ 1:160 (see IMPD for details) are required. The current FDA Guidance (April 2020) recommends neutralizing antibody titers ≥ 1:160. A pilot study in China showed most (39/40) convalescent donors had titers of ≥1:160 (34). A plasma dose of 200 ml is 7% of the total plasma volume for a 60kg individual resulting in a titer reduction to about 1:15 after transfusion into the recipient using 2 CP units with a total volume of 500ml to 650ml would thus be sufficient for most patients. Patients weighing ≥ 150 kg will receive a third unit of CP on day3.

3.3 Timetable of the Study

Recruitment period (months):	6-9
First patient in to last patient out (months):	12
Duration of the entire trial (months):	18
Planned FPI (First Patient in):	01 December 2020
Planned LPO (Last Patient out):	30 November 2021

4 STUDY POPULATION

4.1 Study Population Selection

Approximately 17-69% of individuals infected with SARS-CoV-2 have a moderate/severe course of the disease and must be hospitalized (8). The mortality rate of hospitalized SARS-CoV-2 positive patients in Germany was 22% (8). While the risk of a severe COVID-19 infection for the whole population is rather low, there are individuals with a significantly increased risk of disease progression. These include older individuals (>65 years, especially >80 years), individuals with pre-existing conditions such as COPD, coronary artery disease (CAD), diabetes mellitus, chronic kidney disease (CKD), as well as obese individuals (BMI >35 kg/m^2). Thus, individuals at risk for disease progression may significantly benefit from an early antiviral therapy. The risk factors mentioned above were used to define the target group of the RES-Q HR study.

Table 1 Definition of risk groups

Risk Group	OR hospitalization (WHO ≥ 3)	OR ICU admission (WHO ≥ 5)	OR death (WHO 8)
Age	37.9 (>75 years)		8.5 (>70 years)<br>11.1 (>80 years)
BMI > 40 kg/m2	2.5		
COPD		17.8	
CAD		4.4	
Diabetes mellitus		2.8	
CKD	2.6		
Active tumor disease	3.1		

4.2 Inclusion Criteria

Prior to study participation, a written informed consent will be obtained from each patient according to the local regulatory and legal requirements. Each signature must be dated by each signatory and the informed consent and any additional information form retained by the

investigator as part of the study records. A copy of the informed consent and any additional information must be given to each patient.

Participants must meet all of the following inclusion criteria to be eligible for participation in this study:

1. Individuals (female, male diverse) ≥ 18 years with SARS-CoV-2 infection, confirmed by PCR before study enrollment
2. SARS-CoV-2 positive PCR **≤ 3 days old** (date of NP swab)
3. Presence of ≥ 1 SARS-CoV-2 typical symptom (fever, cough, shortness of breath, sore throat, headache, fatigue, taste disorder, diarrhea, abdominal symptoms, exanthema) and symptom duration ≤ **3 days**.
4. Ability to provide written informed consent
5. Potassium values between 3.5-4.9 mmol/l
6. Platelets on full blood count must be ≥ 50.000/µl
7. Presence of **at least one** of the following criteria:
   - Patients > 75 years
   - Patients > 65 years with at least one other risk factor (BMI >35 kg/m^2, coronary artery disease, CKD with GFR <60 ml/min but ≥ 30 ml/min, diabetes mellitus, active tumor disease)
   - Patients with a BMI >35 kg/m^2 with at least one other risk factor (CAD, CKD with GFR <60 ml/min but ≥ 30 ml/min, diabetes mellitus, active tumor disease)
   - Patients with a BMI >40 kg/m^2
   - Patients with chronic obstructive pulmonary disease (COPD) and/or pulmonary fibrosis

Female participants of childbearing potential who engage in heterosexual intercourse must use at least one acceptable contraceptive measure from screening until 30 days after the last study dose:

- hormonal methods (oral contraceptive, injectable progesterone, subnormal contraceptive implant, transdermal contraceptive patch)
- hormonal intrauterine device
- non-hormonal intrauterine device (IUD)
- bilateral tubal occlusion
- vasectomy in the male partner

Not acceptable birth control methods are:

- periodic abstinence (e.g. calendar, ovulation, symptothermal, post-ovulation methods)
- withdrawal (coitus interrupts)
- spermicides only and
- lactational amenorrhea method.

Female participants must also refrain from egg donation and in vitro fertilization during treatment and until 30 days after last study dose.

A female born participant following onset of puberty until reaching menopause is considered to be of childbearing potential. Women are considered postmenopausal when older than 54 years and cession of previously occurring menses for more than 12 months. Women with permanent sterilization by hysterectomy, or by bilateral oophorectomy, or bilateral salpingectomy are not considered of childbearing potential independent of participant age.

Male participants with female partners of childbearing potential should use condoms when engaging in intercourse of reproductive potential.

4.3 Exclusion Criteria

1. Age <18 years
2. Unable to give informed consent
3. Pregnant women or breast-feeding mothers
4. Previous transfusion reaction or other contraindication to a plasma transfusion
5. Known hypersensitivity to camostat mesylate and/or severe pancreatitis
6. Volume stress due to CP administration would be intolerable
7. Known IgA deficiency
8. Life expectancy < 6 months
9. Duration SARS-CoV-2 typical symptoms > 3 days
10. SARS-CoV-2 PCR detection older than 3 days
11. SARS-CoV-2 associated clinical condition ≥ WHO stage 3 (patients hospitalized for other reasons than COVID-19 may be included if they fulfill all inclusion and none of the exclusion criteria).
12. Previously or currently hospitalized due to SARS-CoV-2
13. Previous antiviral therapy for SARS-CoV-2
14. ALT or AST > 5 x ULN at screening
15. Liver cirrhosis > Child A (patients with Child B/C cirrhosis are excluded from the trial)
16. Chronic kidney disease with GFR < 30 ml/min
17. Concurrent or planned anticancer treatment during trial period
18. Accommodation in an institution due to legal orders (§40(4) AMG).
19. Any psycho-social condition hampering compliance with the study protocol.
20. Evidence of current drug or alcohol abuse.
21. Use of other investigational treatment within 5 half-lives of enrollment is prohibited
22. Previous use of convalescent plasma for COVID-19
23. Concomitant proven influenza A infection
24. Patients with organ or bone marrow transplant in the three months prior to Screening Visit

4.4 Screening Failures

Screening procedures can begin only after informed consent is obtained. After the screening evaluations have been completed, the investigator or designee is to review the inclusion/exclusion criteria and determine the subject's eligibility for the study. Subjects who are found to be ineligible will be told the reason for ineligibility.

Subjects, who are randomized into the CP arm and for whom no suitable CP can be found will be ineligible and thus present as screen failure. The cumulative number of participants for whom no suitable CP will be found are documented as a secondary endpoint. Subjects who are found to be ineligible will be told the reason for ineligibility. Individuals who do not meet the criteria for participation in this study (screen failure) will not be rescreened.

4.5 Strategies for Recruitment and Retention

4.5.1 Recruitment

In order to assure the achievement of the recruitment goal, SARS-CoV-2 test centers in the catchment area of the respective study center will be contacted and patient information flyers will be made available for patients. Since no treatment options for patients fulfilling the inclusion criteria for this study is established, it is expected that patients will report to the study centers and volunteer for study participation. Recruitment efforts will also include dissemination of information about this trial to other medical professionals / hospitals, as well as distribution of information on the study on the homepage of the study centers (download of the flyer via homepage). The catchment area may be extended or targeted according to the current epidemiological situation in the area of the respective study center.

4.5.2 Retention

A high retention rate is assured by comprehensive explanation of the study procedures to patients at baseline and all subsequent visits. Time schedules with all appointments for follow-up visits will be handed out to patients and if patients agree in written form, reminders will be sent as short telephone messages the day before the visit. Patient will have a high motivation to stay in the study since no other treatment is currently available for the study population. The 2:2:1:1 randomisation strategy minimizes the proportion of patients in the SoC and placebo arms. Furthermore, in case of progression of COVID-19, all patients will be eligible to further treatment, such as remdesivir. On the background that novel therapeutic options may arise and that SoC may thus change during the trial, the principal coordinating investigators together with the scientific committee and the DSMB will re-evaluate continuation of the study all 2-3 months.

4.5.3 Compensation Plan for Subjects

Not applicable

4.5.4 Costs

There is no cost to subjects for the research tests, procedures/evaluations and study product while taking part in this trial. Procedures and treatment for clinical care including costs associated with transportation to and from the hospital/outpatient department during the period where the patient is potentially infectious will be billed to the study. In case the

patient’s disease worsens and requires admission to the hospital, the costs associated with hospital stay will be billed to the subject, the subject’s insurance or third party as applicable. At this point the patient will have reached the study endpoint.

5 INVESTIGATIONAL MEDICINAL PRODUCTS

5.1 Convalescent Plasma

5.1.1 Description and Administration

Eligibility of donors for convalescent plasma will be assessed according to the national German Guidelines *“Richtlinie Hämotherapie*”. The procedures for plasmapheresis, frequency and intervals of plasmapheresis procedures, monitoring of repeat donors for serum protein concentration and serum IgG concentration will be performed according to the “*Richtlinie Hämotherapie*”.

In addition to the standard assessments, the titer of neutralizing anti-SARS-CoV-2 antibodies (NT) will be measured in a sample drawn prior to plasmapheresis.

5.1.1.1 Plasmapheresis

The donors will donate either 650 ml, 750 ml or 850 ml of plasma depending on their body weight. Plasmapheresis will be performed according to institutional SOPs.

According to the specification one plasma unit has a volume of 190 ml to 350 ml. The target volume is 250 ml, 283 ml or 325 ml plasma in the RES-Q-HR Trial. This results in the following volumes per unit:

Table 2 Collection volume per plasmapheresis and resulting number and volume of therapeutic units

Donor weight (kg)	Collection volume per plasmapheresis (ml)	Units per plasmapheresis	Volume per unit (ml)
**50 - 60**	650	2	325
**61 - 70**	750	3	250
**> 70**	850	3	283

5.1.1.2 Infectious Disease Marker Testing and CP-Release

In order to provide enough plasma in a reasonable time the quarantine time was canceled as published by the regulatory authorities (PEI). Except for that production of convalescent plasma undergoes the same criteria as plasma products for transfusion purposes – as described in the permission/ license according to AMG § 13.

As described in the permission of the blood donation services high-sensitive PCR for hepatitis virus A, B, C, E, West nile virus (WNV) according to the “*WNV-Stufenplan PEI*” and HIV will be/ was performed.

5.1.1.3 Characterization of CP for this study

The investigational product, HCIP, is anti-SARS-CoV-2 positive convalescent plasma. HCIP will be collected by apheresis from healthy adults identified as having recovered from COVID-19 (see below for donor selection). Potential donors who meet these qualification standards will be/ were referred to a registered blood center where donors will be/ were evaluated according to current blood donation requirements; plasma will then be/ was collected and licensed and frozen within 24 hours of phlebotomy.

The donors for CP must meet the following criteria in accordance with the EU guidance on collection of COVID-19 CP (35):

a. Informed written consent to donate plasma
b. Previous infection with SARS-CoV-2 as documented by either: 1) a positive PCR (from nasal or nasopharyngeal swab, bronchoalveolar lavage or stool) or 2) a past medical history suggestive of COVID-19 and presence of anti-SARS-CoV-2 antibodies (time of first positive test will be documented)
c. Proven to have cleared SARS-CoV-2 from nasopharyngeal mucosa by either: 1) one negative PCR result from nasal swabs or nasopharyngeal swabs and an interval of at least 2 weeks since resolution of SARS-CoV-2 associated symptoms or 2) an interval of at least 4 weeks since resolution of symptoms of SARS-CoV-2 infection. Time and types of symptoms will be documented as well as time of resolution, as well as most severe clinical status according to 8-point ordinal WHO scale.
d. No residual severe organ dysfunction due to COVID-19 (which organ systems were affected as well as the date when last affected will be documented)
e. Negative test for antibodies against HLA class I, class II and HNA-antigens in female donors with a history of pregnancy.
f. Antibodies against SARS-CoV-2 (ratios) at time point of first donation: "Roche" > 15 and / or IgG S1 ("Euroimmune") ≥ 3.5; preferential IgA S1 ("Euroimmune") ≥ 3.5; Abbott IgG CMIA ≥ 3.5
g. Anti-SARS-CoV-2 antibodies detectable in a neutralization assay (NT-titer) of ≥ 1:160 (centrally measured at Institut für Virologie, Düsseldorf, see IMPD for details)

In addition to these criteria study specific eligibility criteria the standard criteria for approval as a plasma donor according to the national German Guidelines ("*Richtlinie Hämotherapie*") have to be observed at each donation:

- Age: first donors 18 - 60 years, repeated donors 18 - 68 years
- Body weight: ≥ 50 kg
- Overall impression: no obvious signs of disease
- Hemoglobin level: women: ≥ 125 g/l, men: ≥ 135 g/l
- Leukocytes, erythrocytes, platelets, mean corpuscular volume: normal
- Total protein (prior to first plasmapheresis and on occasion of every fifth donation): ≥ 60 g/l (Serum)
- IgG (prior to first plasmapheresis and on occasion of every fifth donation): ≥ 6,0 g/l (Serum)
- In case IgG levels are below 6,0 g/L the interval until the next plasmapheresis has to be extended by at least 2 weeks
- Blood pressure: systolic: 100 - 180 mmHg, diastolic: < 100 mm Hg

- Heart rate: rhythmic, rate 50 - 110/min; a heart rate below 50/min might be acceptable for athletes
- Body temperature: < 37.4°C after equilibration in the blood donation center
- Skin (at side of venipuncture): No lesions

For serological testing for SARS-CoV-2 two samples (serum, volume 5-7 ml) will be collected and stored at +4°C in order to perform testing of antibody titers and specificity at each plasmapheresis session for prospective collected products.

For already collected samples SARS-CoV-2 antibodies and NT-titers will be determined from a retained sample. The results of these additional analyses of SARS-CoV-2 antibodies need to be available before release of the CP to the study centers.

5.1.2 Labelling of CP

CP will be provided to the investigators in standard plasma unit bags, which will be labelled additionally as IMP for the RES-Q-HR study as described in the IMPD. CP have to be labelled with a label in addition to their regular labelling as regular blood products according to marketing authorization for FFP from plasmapheresis.

Labelling of all CP units used in the RES-Q HR trial will be carried out in the ITZ Duesseldorf. The additional RES-Q-HR label contains the statement for use in a clinical trial, the study name and the EudraCT number, the name of the sponsor and the name of the lead investigator.

The additional label has the following content:

- Name and address of the sponsor:

  Heinrich-Heine-University Düsseldorf
  Universitätsstraße 1
  D-40225 Düsseldorf

- Phone number of the sponsor: 0211-8104071
- Protocol code: RES-Q-HR
- EudraCT Number: 2020-004695-18
- RES-Q-HR CP Identification Number: (e.g. A 0001; B 0001; AB 0001; O 0001)
- Declaration, that the product is an IMP for the above-mentioned clinical trial.

Further information on the convalescent plasma SARS-CoV-2 will be provided in the Investigational Medicinal Product Dossier (IMPD).

5.1.3 Dosing and Administration of CP

- Plasma (2 units) will be administered within **36 hours** of enrollment. In case a participant weighs ≥ 150 kg, a third unit of CP will be administered on day 3.
- Transfusions will be performed by qualified/skilled personnel in settings equipped to handle potential complications of transfusion. Plasma will be transfused in established hospital or ambulatory settings with transfusion experience.
- Infusion rate will be 500 ml/hour and patients will be monitored for at least 1 hour after transfusion before being discharged.
- Duration of transfusion should be 30 to 60 minutes but must not exceed 4 hours.
- Medicines to minimize mild transfusion reactions during occurrence (e.g. acetaminophen, diphenhydramine) may be given at the discretion of the investigator.

- If an AE develops during infusion, the infusion may be slowed or stopped as per investigator’s decision.
- Classification of transfusion-associated AE will follow the CDC National Healthcare Safety Network Biovigilance Component Hemovigilance Module Surveillance Protocol20 (https://www.cdc.gov/nhsn/pdfs/biovigilance/bv-hv-protocol-current.pdf).
- Management of transfusion-associated AE will follow the current German transfusion guidelines; Outside of a simple allergic transfusion reaction, the transfusion will be discontinued and investigated appropriately (i.e. per standard practice guidelines).

Infusion of plasma will be halted if any of the following manifestations of anaphylaxis develop and will not be restarted:

- Skin or mucous membrane manifestations: hives, pruritus, flushing, swollen lips, tongue or uvula
- Respiratory compromise: dyspnea, wheezing, stridor, hypoxemia
- Transfusion related volume overload
- A decrease in systolic blood pressure to < 90 mmHg or >30% decrease from baseline or a diastolic drop of >30% from baseline.
- Tachycardia with an increase in resting heart rate to > 130 bpm; or bradycardia <40 that is associated with dizziness, nausea or feeling faint
- Syncope
- Confusion
- Any other symptom or sign which in the good clinical judgment of the study clinician or supervising physician warrants halting the infusion. For example, the rapid onset of gastrointestinal symptoms, such as nausea, vomiting, diarrhea, and cramps, for instance, may be manifestations of anaphylaxis and may warrant an immediate halt prior to meeting full SAE criteria

There is no dose modification planned. If a *transfusion reaction* is suspected early discontinuation of plasma transfusion can result in a lower dose of convalescent plasma administered.

5.1.4 Allocation and Transfusion of CP from a Donor to a Study Participant

A central virtual convalescent database/plasma bank will be established at the ITZ in Duesseldorf. All IMPs will be tested for anti-SARS-CoV-2 neutralizing antibodies centrally in Duesseldorf and labelled as IMP. All IMPs will be entered into the CP database.

CPs will be distributed to the “*Transfusionzentren*” of the different study centers after initiation of the respective center together with the “CP list” for this center. Blood groups will be determined for all participants of the trial. Once the patient is randomized into the CP arm, the investigator will ask for the IMP at his/her local blood bank/transfusion service, which will then distribute the respective CP according to the blood group and CP list.

A copy of the IMP dispatch note will be faxed to

Koordinierungszentrum für Klinische Studien Düsseldorf

Fax: 0211-81-19705

and documented into the eCRF and also documented into the patient’s health record according to the national regulations. Infusion start time, end time, outcome and any adverse

events encountered during the infusion will be documented on the CP transfusion chart, in the eCRF as well as in the patient's health record.

The investigator and local blood bank at the center will be responsible for accountability. The ITZ Duesseldorf will be responsible for the overall accountability of the IMPs.

There will be no return of products from the clinical center back to the manufacturer/local blood bank/transfusion center.

CPs shall be transfused within 1 hour after thawing. Time of start of thawing, start of transfusion and end of transfusion has to be recorded for each CP unit. CPs must be transfused within 6 hours. CPs thawed but not transfused will be discarded. This will be documented both at the local blood bank, in the eCRF and the local as well as central plasma data base.

Allocation of convalescent plasma from a donor to COVID-19 patient:

Each patient shall receive both CP transfusions from a single donor if the availability of CP units allows this one-to-one assignment between a COVID-19 patient and an ABO-identical or compatible donor. However, strict adherence to the treatment schedule as defined in this clinical trial protocol has priority over a one-to-one assignment between a patient and a particular donor of CP.

This rule of a one-to-one assignment, provided that availability of plasma units allows this approach, shall

i) minimize donor exposure and therefore risk of transmitting infectious agents and risk of allergic reactions and

ii) facilitate the analysis whether donor factors (e.g. antibody specificity, titer of antibodies) correlate with response.

Blood group compatibility:

FFPs/CP shall be transfused ABO-identical. If availability of convalescent plasma does not allow ABO-identical transfusion, a compatible unit of CP according to the following table will be transfused.

Table 3 Transfusion therapy with CP in SARS-CoV-2: selection of the ABO phenotype of units to transfuse

ABO phenotype of the recipient	ABO phenotype of convalescent plasma units (in order of preference)
**0**	O, A, B, AB
**A**	A, AB
**B**	B, AB
**AB**	AB

If a transfusion reaction is suspected early discontinuation of plasma transfusion can result in a lower dose of convalescent plasma administered.

5.1.5 Accountability, Product Storage, Stability and Destruction

Central recording: All CP units, either collected in Duesseldorf or bought from other licensed blood banks, will be characterized, labelled and distributed centrally through the ITZ Duesseldorf. Thus, all CPs for this trial will be recorded in the central database. Distribution to the different centers and the different participants will also be documented in the central database. Information for locally administered CPs will be reported to the ITZ via Fax as well as through the eCRF forms.

Local recording: A list with all IMP-CPs will be distributed to the local blood bank together with the CPs. The allocation of a specific CP to the study participant will be determined via the "Center-CP list" in ascending order. The allocation will be recorded locally as well.

Per patient recording: According to transfusion law the transfusion of CP to a specific study participant will be documented in the patients' health record. The RES-Q-HR specific CP-product code will also be recorded in the eCRF of the patient and on the transfusion chart.

5.1.6 Product Storage, Stability and Destruction

Plasma units can be stored according to the license of the manufacturer (1-2 years) and then are destroyed according to local guidelines.

5.2 Camostat Mesylate

5.2.1 Description

SARS-CoV-2 entry into epithelial cells of the respiratory tract is dependent on the angiotensin-converting enzyme 2 (ACE 2) receptor and priming of the SARS-CoV-2 S protein by the host cell serine protease TMPRSS2 (21, 28). Inhibition of the serine protease activity of TMPRSS2 with camostat mesylate efficiently blocked entry of SARS-CoV-2 into the lung cell line Calu-3 as well as into primary human lung cells without significant cytotoxic side effects ($EC_{50} \approx 1\mu M$) (21). Camostat mesylate has been shown to block TMPRSS2 activity (21, 29, 36) and has been approved in Japan for chronic pancreatitis in a dose of 600 mg daily (clinical information Foipan®).

Camostat mesylate *in vitro* exhibits a potent inhibitory effect on trypsin, plasma kallikrein, plasmin, thrombin, $C_{1\text{-}r}$- and C_1-esterase (37). An active metabolite in blood, 4-(4-guanidinobenzoyloxy) phenylacetate after oral administration of camostat mesylate, is nearly equipotent with the mother compound in these inhibitory activities (*in vitro*).

Camostat mesylate, after oral administration, acts promptly on kinin formation, fibrinolytic, coagulation and complementary systems rapidly inhibiting enzyme activities and their abnormal increases. This results in remission of inflammatory symptoms and pain as well as in improvement of serum amylase level in chronic pancreatitis (clinical information Foipan®).

In vitro camostat mesylate and its metabolite 4-(4-guanidinobenzoyloxy) phenylacetate did not inhibit CYP1A2, CYP2C9, CYP2C19, CYP2D6 and CYP3A4 (clinical information Foipan®).

5.2.2 Formulation, Labelling and Packaging of Camostat Mesylate and Matching Placebo

Camostat Mesylate

The supplied IMP camostat mesylate tablets are blinded by capsule overcapping to be identical in physical appearance to the placebo. It contains Hydroxypropylmethylcellulose, Carmellose calcium, Magnesium stearate, Polyoxyethylene(105)polyoxypropylene(5)glycol, Lactose hydrate as inactive ingredients.

Matching Placebo

The supplied placebo tablets are blinded by capsule overcapping to be identical in physical appearance to the IMP camostat mesylate. The placebo differs in inactive ingredients and contains lactose-monohydrate, cellulose, magnesium stearate (Ph. Eur.) and microcrystalline cellulose.

Each of the study products will be labelled according to manufacturer specifications and include the statement "For Investigational Use only."

5.2.3 Dosing and Administration

The dose of camostat mesylate is 200 mg three times daily for 7 days. Capsule containing the camostat mesylate is swallowed with water or any other liquid and can be taken with or without food. Patients are monitored for 1 hour after taking the first dose of camostat mesylate in order to recognise signs of hypersensitivity. In case of hypersensitivity or any other adverse reaction, appropriate measures will be immediately taken according to the judgement of the study physician.

Camostat Mesylate

- Days 1 – 7: 200 mg p.o. 1-1-1

Placebo

- Days 1 – 7: 200 mg p.o. 1-1-1

A matching placebo will be given at an equal volume at the same schedule.

See the protocol-specific Manual of Procedures (MOP) Appendices for detailed information on the preparation, labelling, storage, and administration of camostat mesylate and placebo. All missed doses are not made up.

No dose modifications are planned; all missed doses are not made up. In case of hypersensitivity is suspected, treatment needs to be stopped and discontinued.

5.2.4 Accountability, Product Storage, Stability and Destruction

Accountability

Camostat mesylate: The site PI is responsible for study product distribution and disposition and has ultimate responsibility for study product accountability. The site PI may delegate to the participating site's research pharmacist responsibility for study product accountability.

The participating site's research pharmacist will be responsible for maintaining complete records and documentation of study product receipt, accountability, dispensation, storage conditions, and final disposition of the study product(s). Time of study drug administration to the subject will be recorded on the appropriate data collection form (CRF).

All study product(s), whether administered or not, must be documented on the appropriate study product accountability record or dispensing log. The sponsor's monitoring staff will verify the participating site's study product accountability records and dispensing logs per the site monitoring plan. Refer to the protocol-specific MOP for details on storing active and placebo medications.

Product storage, stability and destruction

Store at room temperature (15-25°C) in original press-through package. Protect from light. The expiration date is indicated on the package (1 year).

After the study treatment period has ended or as appropriate over the course of the study after study product accountability has been performed and after sponsor's approval, unused camostat mesylate and placebo will be shipped to the Apotheke des Universitätsklinikums Düsseldorf for destruction.

5.3 Treatment Compliance

CP will be administered by the investigator on site. The first dose of camostat mesylate or placebo will be administered by a member of the clinical research team, that is qualified and licensed to administer the study product. Administration will be documented in electronic case report including the date, time, IMP identification number and for CP additionally the site of injection.

5.4 Concomitant Medication / Concomitant Therapy

The IMP, CP and camostat mesylate, each in addition to Standard of Care (SoC) will be compared to the respective control groups with SoC alone (for CP) or SoC plus placebo (camostat mesylate). SoC comprises the supportive care according to the current national guidelines and recommendations. During the trial period, SoC **excludes other experimental antiviral therapies**, unless the patient's clinical status deteriorates. In this case the participant has reached the study endpoint (clinical stage 4b) and can receive any available therapeutic strategy. The use of other experimental substances, and/or the participation in other interventional trials, is not permitted. Concomitant anticancer treatment is not allowed up to 72 hours before screening as well as during IMP treatment and up to 14 days after last dose of IMP (e.g. day 21 in Camostat mesylate/placebo group).

Antiviral strategies approved during the running trial will be evaluated by the PCI and DSMB to determine whether these therapeutic/intervention should made available to all subjects in the trial as part of SoC or whether the trial should be terminated early. Concomitant medications administered up to day 90 are recorded. No clinically significant interactions with concomitant medications are reported to date and/or expected.

5.5 Rescue Medicine and Procedures in Case of Emergency

Specific rescue medicine is not available. However, if an allergic reaction occurs, patients will be treated according to the guidelines for allergic reactions and according to the respective SOPs of each center.

5.6 Blinding Procedures and Unblinding

The Apotheke des Universitätsklinikums Düsseldorf is responsible for blinding, labelling and delivery of the per oral IMP to the trial sites. A double-dummy approach is used: The supplied camostat mesylate and placebo tablets are blinded by capsule overcapping to be identical in physical appearance to the IMP.

For unblinding, the centers receive opaque rescue envelopes for each participant randomized to the camostat mesylate / placebo + SoC arm of the trial. Each envelope is assigned to a specific subject number and includes the information, if the participant received either verum or placebo.

6 STUDY PROCEDURES

6.1 Recruitment / Screening Procedures

Entry into screening does not guarantee enrollment into the study. Screening and study visits **(not CP infusion**) may be performed in an outpatient setting, in an inpatient setting or at the participant's home (not CP infusion) depending on the health status of the patient and the preference of the center. Transport of SARS-CoV-2 PCR positive, infectious patients to the outpatient department/study center must be carried out according to current hygiene and quarantine recommendations ("Infektionstransport"). Patients will be educated on the contagious nature of COVID-19, hygiene guidelines and quarantine/self-isolation measures according to the RKI recommendations.

6.2 Methods of Obtaining Informed Consent

In order to enrol a subject into the trial, an informed consent will be obtained from each individual participating in this study prior to any trial related interventions.

First, patients will be informed in written and oral form regarding the key facts of the study, the procedures that will follow, and the reasonably foreseeable risks or discomforts as well as potential benefits. They will also be informed of potentially alternative "experimental" treatments besides the trial. As a second step, an authorised member of the trial team will give each participant an informed consent document with details about the study including its purpose, duration, procedures, and key contacts, as well as risks and potential benefits. The patients then will decide whether to give written and oral consent. Patients unwilling to consent will not be included in the study.

6.3 Methods of Avoiding Simultaneous Enrolment in Other Trials

The patient information sheet points out that there is no possibility of participating in other clinical trials investigating an experimental therapy for COVID-19 at the same time. The patient will be informed about this issue in the interview with the investigator and it is part of the informed consent form.

6.4 Enrolment and Randomisation (Assignment of Study Medication)

Patients, who meet all inclusion criteria and who have given their written informed consent will be reported to the sponsor with the following data.

List of data provided for enrolment and randomization:

- Name and address of the responsible trial center/ institution
- Name of person responsible for clinical trial or contact person
- Telephone / fax numbers
- Times, at which trial center can be contacted
- Study site, Investigator
- Pseudonym of patients who need randomization
- Gender

6.5 Measures to Minimize Bias: Randomization and Blinding

This study is a randomized, controlled trial to evaluate the safety and efficacy of two therapies: CP and camostat mesylate, against their specific control groups. The camostat mesylate and its placebo group will be double blinded while the CP and its placebo will be open label.

The study is a multicenter trial that will be conducted in approx. 10 – 15 centers in Germany. At each center, patients will be randomized into four groups: two treatment groups and two control groups. The randomization rate in this study is two to one (2:1) in favor to therapy, i.e. included patients have twice the chance to receive interventional therapy than placebo / SoC.

6.6 Clinical Examinations and Trial-related Deviations from Clinical Practice

A schedule of events is given in appendix 14.1. Screening and study visits may be performed in an outpatient setting, clinical (inpatient) setting or at the participant's home **(except CP infusion**) depending on health status of the patient and the preference of the center.

Participants will be screened within 24 hours prior to randomization to determine eligibility for participation in the study.

6.6.1 Pre-trial Examinations (Screening / Inclusion Examination)

Participants will be screened within 24 hours prior to randomization to determine eligibility for participation in the study.

At the screening visit the following will be performed and documented

- Obtain written informed consent
- Obtain a medical history including the following information:
  - Date of first SARS-CoV-2 related symptoms (duration ≤3 days)
  - Overall SARS-CoV-2 related symptoms (which, duration, severity)
  - Prior (last 8 weeks) and current medication
  - Known allergies
  - Past medical history
  - Demographics

- Complete physical examination (urogenital and anorectal exam not required), including vital signs (temperature, heart rate, blood pressure), body weight and body height
- ECG
- Respiratory status including: respiratory rate, SpO_2 on room air
- Clinical status on 8-point modified WHO ordinal scale
- Blood group
- Hematology (CBC and differential)
- Laboratory values: Na, K, glucose, Crea, BUN, AST, ALT, GGT, ALP, bilirubin, PT, INR, CRP, PCT, Troponin, CK, NT-proBNP, D-Dimers, ferritin, fibrinogen
- Baseline anti-HCV, anti-HIV, HBs-Antigen, anti-HBc IgG, anti-HEV
- IL-6 serum levels
- SARS-CoV-2 PCR from nasopharyngeal swab
- Anti-SARS-CoV-2 antibody titers (determined by either Euroimmune, Roche or Abbott commercially available tests)
- Optional sampling for exploratory biomarkers (including: 2.5 ml PAXgene®, 3 x 10 ml EDTA, 1 x 4 ml EDTA, 2 x 8 ml serum, 1 x 8 ml plasma, 25 ml urine)
- Women of potential childbearing age will need a urine pregnancy test on site
- Documentation of SARS-CoV-2 PCR ≤ 3 days prior to screening
- Record all SAEs and all protocol related AEs occurring after obtaining written consent
- SF-12 quality of life questionnaire and Visual analogue scale (VAS)

Participants meeting all of the inclusion criteria and none of the exclusion criteria may be enrolled on the same day as screening for randomization into the study. Participants may also receive the first dose of IMP after randomization on the same day (day 0) or within 36 hours.

6.6.2 Examinations During Trial

Treatment assessment on day 1

Depending on preference of the investigator and availability of CP the day 1 baseline visit can be carried out in combination with the screening. In this case, following randomization the IMP will need to be administered and the patient will be supervised for another hour after IMP administration.

In case day 1 is not combined with the screening visit the following evaluations have to be carried out on day 1:

- Review and documentation of concomitant medication
- Physical examination (as above)
- Vital signs: temperature, heart rate, blood pressure
- Respiratory status including: respiratory rate, SpO_2 on room air
- Clinical status on 8-point modified WHO ordinal scale
- Adverse events/serious adverse events

Administer IMP (CP, camostat mesylate, placebo) according to the result from randomization

Treatment assessment on days 3, 5, 8 and 14

- Complete physical examination (urogenital and anorectal exam not required), including vital signs (temperature, heart rate, blood pressure), body weight and body height
- Respiratory status including: respiratory rate, SpO_2 on room air
- Clinical status on 8-point modified WHO ordinal scale
- Hematology (CBC, differential blood count)
- Laboratory values: Na, K, glucose, Crea, BUN, AST, ALT, GGT, ALP, bilirubin, PT, INR, CRP, PCT, Troponin, CK, NT-proBNP, D-Dimers, ferritin, fibrinogen
- Women of childbearing age will have an additional urine pregnancy test on d8
- IL-6 serum levels
- SARS-CoV-2 PCR from nasopharyngeal swab
- Anti-SARS-CoV-2 antibody titers (determined by either Euroimmune, Roche or Abbott commercially available tests)
- Optional sampling for exploratory biomarkers (including: 2.5 ml PAXgene®, 2 x 10 ml EDTA, 2 x 8 ml serum, 1 x 8 ml plasma, 25 ml urine, 10 ml plasma for camostat plasma levels in the respective subgroup on day 3 and day 8)
- Record all SAEs and all AEs occurring
- Review and documentation of concomitant medication
- On day 8 a second ECG will be recorded

Treatment assessment on Days 28 and 56 (telephone interview)

- Clinical status on 8-point modified WHO ordinal scale
- Review and documentation of concomitant medication
- Record all SAEs and all AEs occurring after obtaining written consent
- Day 28 only: SF-12 quality of life questionnaire, VAS

6.6.3 Final Examination (day 90 ± 5 days)

- Complete physical examination (urogenital and anorectal exam not required), including vital signs (temperature, heart rate, blood pressure), body weight and body height
- Respiratory status including: respiratory rate, SpO_2 on room air
- Clinical status on 8-point modified WHO ordinal scale
- Hematology (CBC and differential)
- Laboratory values: Na, K, glucose, Crea, BUN, AST, ALT, GGT, ALP, bilirubin, PT, INR, CRP, PCT, Troponin, CK, NT-proBNP, D-Dimers, ferritin, fibrinogen
  anti-HCV, anti-HIV, HBs-Antigen, anti-HBc IgG, anti-HEV
- IL-6 serum levels
- SARS-CoV-2 PCR from nasopharyngeal swab
- Anti-SARS-CoV-2 antibody titers (determined by either Euroimmune, Roche or Abbott commercially available tests)
- Optional sampling for exploratory biomarkers (including: 2.5 ml PAXgene®, 2 x 10 ml EDTA, 1x 4 ml EDTA, 2 x 8 ml serum, 1 x 8 ml plasma, 25 ml urine)
- Record all SAEs and all AEs
- SF-12 quality of life questionnaire, VAS

6.6.4 Exploratory Assessments (optional)

Exploratory endpoints of the study are optional (additional patient information and consent form) and as follows:

- Plasma concentrations of camostat mesylate (d3 40-45 min after dosing, d8)
- Total immunoglobulin titer in serum of all study participants
- Baseline levels and change from baseline for inflammation/immune related, ARDS associated, coagulation related biomarkers including chemokine, cytokine, ACE and ACE cleavage products, miRNAs, SuPAR, etc. in serum / plasma
- Baseline and changes from baseline in cellular immunophenotypes (whole blood analyses, PAXgene®, RNASeq, etc)
- Baseline and change from baseline urine biomarkers
- Analysis of host microbiome (from NP swabs)
- Emergence of viral resistance
- Presence of co-infections
- Host genomics – in addition to the study-specific informed consent, participants will be asked to give informed consent and contribute a sample for additional genomic research. Samples collected for optional genomic research will be destroyed no later than 15 years after the end of the study.

7 DISCONTINUATION AND WITHDRAWAL

7.1 Individual Halting

Stopping rules for the individual patient with omission from the trial are:

- Withdrawal of consent, in this case the patient leaves the trial and no follow up visits will be carried out
- Death of the patient
- Severe protocol violations, administrative problems or non-compliance with the study protocol by the patient incompatible with further participation in the study

Patients are free to withdraw from participation in the study at any time upon request, without any consequence. Patients should be listed as having withdrawn consent only when they no longer wish to participate in the study and no longer authorize the investigators to make efforts to continue to obtain their outcome data.

Stopping rules for the individual patient’s therapy, but patient remains in trial and receives follow up visits according to protocol if possible

- an increase in ALT and AST values >5x ULN and /or a 3-fold increase from baseline values
- an increase in bilirubin >5x ULN, an increase in AST and ALT >5x ULN and /or a 3-fold increase from baseline values and rise in bilirubin 3x baseline level.
- Reduction in GFR <30ml/min
- The occurrence of any medical condition that the investigator or sponsor determines may jeopardize the patient’s safety if he/she continues with the trial.
- Clinical signs of anaphylaxis /hypersensitivity
- Hyperkalemia (≥5.0 mmol/l) after pre-analytical error is excluded (e.g. hemolysis)

- Thrombocytopenia < 50.000/µl
- Patient requests to discontinue study drugs
- Any serious adverse event (SAE), clinically significant adverse event, severe laboratory abnormality, intercurrent illness, or other medical condition that indicates to the investigator that continued participation is not in the best interest of the patient
- Patient fails to comply with protocol requirements or study-related procedures
- Subject is found to be pregnant after randomization
- Termination of the study with stopping of IMP administration, but further follow up for safety and outcome

Patients, who deteriorate on the 8-point ordinal clinical status scale, which is expected due to the known clinical characteristics of COVID-19, will be eligible to receive remdesivir as deemed beneficial by the investigator.

For an individual subject, CP infusion must be stopped if they have a suspected drug-related event of hypersensitivity (Grade 2 or higher) during the infusion. Subjects who have an IV infusion stopped for a safety related issued will not continue with the second unit of CP.

Occurrence of ≥ grade 3 AE and any serious adverse event (SAE) and suspected to be related to the study medication/CP according to the assessment of the local principal investigator accordance with the PCIs.

Patients who withdraw from this study or are lost to follow-up after signing the informed consent form (ICF) and administration of the study product, will not be replaced. The reason for patient discontinuation from the study will be recorded on the appropriate case report form.

7.1.1 Premature Termination of the Clinical Study

The study will be discontinued if the Sponsor or its designee judges it necessary for medical, safety, regulatory, or other reasons consistent with applicable laws, regulations, and GCP guidelines.

The following events, if applicable, may cause premature termination of the clinical study:

- Early evidence of overt inferiority of the treatment according to the recommendation of the DSMB (decision taken by the Sponsor).
- Emerging data that another therapeutic agent is overtly superior to any other reported intervention and the agent will be available for the patients in the current trial.
- Unjustifiable risk and/or toxicity in risk-benefit analysis (decision taken by the Sponsor), e.g. when adverse events occur, unknown to date with respect to their nature, severity, duration or frequency in relation to the currently established safety profile (substantial changes to the risk-benefit ratio), and therefore medical and/or ethical reasons affect the continuation of the study.
- New scientific evidence provided during the study that could affect the patient's safety (benefit-risk analysis no longer positive).
- Request of the Sponsor or regulatory agency

7.2 Lost to Follow-Up

A subject will be considered lost to follow-up if he or she fails to respond to the telephone follow-up assessment and cannot be contacted with good effort. These efforts will be documented in the subject's record.

Patients who withdraw from this study or are lost to follow-up after signing the informed consent form (ICF) and administration of the study product, will not be replaced. The reason for patient discontinuation from the study will be recorded on the appropriate case report form (CRF).

7.3 Follow-up and Continuing Treatment after Regular Termination

After completing all the protocol treatment and visits, patients will continue with regular visits according to usual practice

In the case of premature termination, the reason for withdrawal must be entered on the appropriate case report form (CRF) page and the patient should be followed for safety and efficacy until 90 days after the last administration of the investigational product.

8 SAFETY

8.1 Definition of Adverse Events and Serious Adverse Events

8.1.1 Adverse Event and Adverse Drug Reaction

An adverse event (AE) is any untoward medical occurrence in a clinical trial subject administered an IMP. There does not necessarily have to be a causal relationship with the IMP. An adverse event can therefore be any unfavourable and unintended sign (including an abnormal laboratory finding), symptom or disease that occurs during the course of the study, whether or not considered related to the IMP.

AEs include:

- Exacerbation of a pre-existing disease
- Increase in frequency or intensity of a pre-existing episodic disease or medical condition
- Disease or medical condition detected or diagnosed after IMP administration even though it may have been present prior to the start of the study
- Continuous persistent disease or symptoms present at baseline that worsen following the start of the study
- Events considered by the investigator to be related to study-mandated procedures
- Abnormal assessments, (e.g. pathological blood pressure), must be reported as AE, if they represent a clinically significant finding that was not present at baseline or worsened during the course of the study
- Laboratory test abnormalities must be reported as AE if they represent a clinically significant finding, symptomatic or not, which was not present at baseline or worsened during the course of the study or led to dose reduction, interruption or permanent discontinuation of the study drug

AEs do not include:

- Standard monitoring of a pre-existing disease or medical condition that do not worsen e.g., hospitalization for coronary angiography in a patient with stable angina pectoris.
- Hospitalizations for cosmetic elective surgery, social and/or convenience reasons.
- Laboratory test abnormalities must not be reported as AE if they do not represent a clinically significant finding.

An adverse drug reaction (ADR) is any noxious and unintended responses to a study drug related to any dose administered.

8.1.2 Serious Adverse Event and Serious Adverse Drug Reaction

A serious adverse event (SAE) or serious adverse drug reaction (SADR) is any AE that:

- Results in death
- Is life-threatening (i.e., in the opinion of the Investigator(s) the subject is at immediate risk of death from the AE)
- Requires inpatient hospitalization or prolongation of existing hospitalization
- Results in persistent or significant disability/incapacity (a substantial disruption of the subject's ability to conduct normal life functions)
- Is a congenital anomaly/birth defect
- Constitutes an important medical event

Important medical events are defined as those occurrences that may not be immediately life threatening or result in death, hospitalization, or disability, but may jeopardize the subject or require medical or surgical intervention to prevent one of the other outcomes listed above. Medical and scientific judgment should be exercised in deciding whether such an AE should be considered serious.

8.1.3 Suspected Unexpected Serious Adverse Reactions

A suspected unexpected serious adverse reaction (SUSAR) is an adverse event the nature or severity of which is not consistent with the product information available for the IMP, is regarded as serious, and has at least a possible causal relationship with the IMP.

8.2 Severity / Intensity

For both AEs and SAEs, the investigator(s) must assess the severity/intensity of the event. The severity of adverse events (AEs) will be graded on a scale of 1 to 5 according to the National Cancer Institute (NCI) Common Terminology Criteria for Adverse Events Version 5.0 (NCI CTCAE). The NCI CTCAE v5.0 can be viewed online at the following NCI web site:

https://ctep.cancer.gov/protocolDevelopment/electronic_applications/docs/CTCAE_v5_Quick_Reference_8.5x11.pdf.

8.3 Causality

The investigator must assess every AE/SAE whether a causal relationship with the IMP and/or study procedure can be assumed or not. The assessment includes consideration of the nature and type of reaction, the temporal relationship with the IMP, the clinical status of the trial subject, concomitant medication and other relevant clinical factors. If the event is considered

due to lack of efficacy or as a symptom or sign of the underlying disorder, no causal relationship will be assumed.

To assess causality between administration of the investigational product and the Adverse Event the following definitions apply:

- Definitely related:

  The reaction comprehensively follows the administration of the investigational product in the right timeframe or can be measured in body tissues or fluids or represents a known or expected response to the study medication or disappears after discontinuation or dose reduction and reoccurs after re-exposure.

- Probably related:

  The reaction comprehensively follows the application of the investigational product in the right timeframe or represents a known or expected response to the study medication or disappears after discontinuation or dose reduction and cannot be explained by known characteristics of the patient's disease.

- Possibly related:

  The reaction comprehensively follows the application of the investigational product in the right timeframe or represents a known or expected response to the study medication, but could easily be caused by other factors.

- Unlikely:

  Event or laboratory test abnormality, with a time to drug intake that makes a relationship improbable (but not impossible); disease or other drugs provide plausible explanations

- Not related:

  Adequate Information supporting the assumption that there is no causality

- Cannot be evaluated:

  The causality cannot be determined.

Out of these five categories, "**cannot be evaluated**", "**possibly**", "**probably**" and "**definitely**" related to a medicinal product qualify as **adverse reactions**. "**Unlikely**" and "**not related**" do not qualify as a reasonable causal relationship.

8.4 Documentation and follow-up of AEs and SAEs

All Serious Adverse Events (SAEs) and all Adverse Events (AEs) need to be documented, no matter if the Investigator suspects a causal connection to the investigational product or not. The documentation needs to include the following:

- type of event
- start
- duration
- severity and

- causality

Related signs symptoms and laboratory changes should be summarized to a specific disease. The event will be recorded in the CRF. SAEs need to be documented on a separate SAE form. Out of normal range laboratory data need to be analysed concerning their clinical relevance by the Investigator and, if relevant, documented as an AE itself.

All adverse events need to be followed until they subside or stabilize.

The Sponsor will carefully document all SAEs reported by the Investigator. His documentation will be sent to the relevant regulatory authorities.

8.5 Reporting of Serious Adverse Events, pregnancies and changes in risk-benefit assessment

Regardless of the assumed causal relationship, every SAE that occurs during the trial must be documented in the appropriate part of the CRF and on a SAE sheet sent to KKSD as delegate of the sponsor. SAE reporting will be done for each study patient starting with the date where informed consent is given until follow-up visit on day 90 ± 5 days.

The SAE report form must be sent to KKSD via Fax within 24h of becoming aware of the event (see section 12.5.1).

8.5.1 Notification of the Sponsor by the Investigator

The investigator will inform the KKSD about the occurrence of an SAE without delay, at the latest 24 hours after being made aware of the SAE, by sending the SAE report form via fax to:

Koordinierungszentrum für Klinische Studien Düsseldorf

Fax: 0211- 81-19705

The SAE report by the investigator to the sponsor or his delegate shall be followed by detailed, written follow-up reports using the SAE report form.

All SAE will be evaluated by the PCIs, irrespective of whether they are regarded as unexpected and the criteria for the existence of a SUSAR are met.

8.5.2 Assessment of the Serious Adverse Events by the Sponsor

All cases of SAE are assessed by the sponsor with regard to seriousness (s. Section 12.1.2), causality (s. Section 12.3) and expectedness (see sections 12.1.3), regardless of the investigator's assessment. If an AE is "serious", at least "cannot be evaluated", "possibly related" and "unexpected", the criteria for a suspected unexpected serious adverse reaction (SUSAR) are fulfilled.

In case of the occurrence of a SUSAR, it is the responsibility of the sponsor of the study to report these to the national competent authority (BfArM), the leading Ethics Committee as well as to the investigators of the trial – in line with the GCP-Verordnung (GCP-V).

8.5.3 Notification of National Competent Authority, Leading Ethics Committee, and Trial Sites

In case of the occurrence of a SUSAR, it is the responsibility of the sponsor of the study to report these to the national competent authorities (BfArM, PEI), the leading Ethics Committee as well as to the investigators of the trial – in line with the GCP-Verordnung (GCP-V).

- Fatal and life-threatening SUSARs

  The national competent authorities and the leading Ethics Committee must be informed by the sponsor of all fatal or life-threatening SUSARs. This must be done without delay, at the latest 7 calendar days after becoming aware of the minimum criteria for reporting. In all cases, attempts must be made to obtain further relevant information which must be supplied to the national competent authorities and the leading Ethics Committee within a further 8 days.

- SUSARs that are not fatal or life-threatening

  The national competent authority and the leading Ethics Committee will be informed without delay by the sponsor of all SUSARs, at the latest within 15 calendar days of becoming aware of the minimum criteria for reporting. Further relevant details will be passed on as soon as possible. If the information at the time of reporting is incomplete, further information to enable adequate assessment of the case will be requested from the reporter or other available sources.

All investigators at every site will be informed about the SUSAR and all pregnancy cases including the information reported to the BfArM and leading Ethics Committee within the same time frame legally required. If new information is gained, which differs from the scientific information given to the investigators before (e.g. via the investigator's brochure), then the sponsor will provide the new information to all investigators.

8.5.4 Evaluation and Report of Changes in Risk/Benefit Ratio

Without delay and at the latest within 15 days of the decision for the need to do so, the sponsor will inform the BfArM and the leading Ethics Committee about any events or factors implying that the risk-benefit ratio of the IMP has to be reviewed. These especially are:

- Individual reports of expected SADR with an unexpected outcome
- A clinically relevant increase in the rate of occurrence of expected SADR
- SUSAR in trial patients who have already completed the follow-up period of the clinical trial
- Factors emerging in connection with trial conduct or the development of the IMP that may affect the safety of persons concerned

8.6 Pregnancies and Investigator Reporting Responsibilities

8.6.1 Female of Childbearing Potential

For definition of childbearing potential and methods of birth control refer to chapter 4.1. Pregnancies and suspected pregnancies (including a positive pregnancy test regardless of age or disease state) of a female subject occurring while the subject is on study drug or within follow-up period are considered immediately reportable events. Study drug is to be

discontinued immediately and the subject instructed by the investigator to return any unused portion of the study drug to the trial site. The pregnancy, suspected pregnancy, or positive pregnancy test must be reported to the sponsor by sending the Pregnancy Reporting Form immediately via fax to:

Koordinierungszentrum für Klinische Studien Düsseldorf

Fax: 0211-81-19705

The female participant should be referred by the investigator to a physician specialized or experienced in teratology for further evaluation and counseling. The investigator(s) will follow the female subject until completion of the pregnancy and must notify the sponsor of the outcome of the pregnancy as a follow-up to the initial report.

If the outcome of the pregnancy meets the criteria for immediate classification as a SAE (i.e., spontaneous or therapeutic abortion [any congenital anomaly detected in an aborted fetus is to be documented], stillbirth, neonatal death, or congenital anomaly [including that in an aborted fetus]), the investigator(s) should follow the procedures for reporting SAEs. In the case of a live "normal" birth the sponsor should be informed via fax within 24 hours of the investigator's knowledge of the event.

All neonatal deaths that occur within 30 days of birth should be reported, without regard to causality, as SAEs. In addition, any infant death after 30 days that the Investigator(s) suspects is related to the in-utero exposure to the study drug should also be reported by fax within 24 hours of the Investigators' knowledge of the event.

In order to ensure data protection compliance, follow-up reports concerning the outcome of the pregnancy and the medical data of the newborn as well as corresponding SAE reports may only be forwarded to the sponsor with the written consent of all persons who have custody of the child. If the female is found not to be pregnant, any determination regarding the subject's continued participation in the study will be determined by the Investigator(s).

8.6.2 Male Subject

Not applicable

8.7 Development Safety Update Report

Once per year or on request, the sponsor will supply a report on the safety of trial patients with all available relevant information concerning patient safety during the reference period to the national competent authority. This report will also be supplied to the leading Ethics Committee. The development safety update report (DSUR) will be compiled by the sponsor according to the corresponding ICH guideline E2F „Development Safety Update Report – DSUR". The data lock point for the patient data to be included and analyzed is following the date of the study approval from the national competent authority. The sponsor will supply the first report within 60 days of one year after the reference date (data-lock point).

8.8 Data Safety Monitoring Committee

An independent Data Safety Monitoring Committee (DSMB) will oversee the trial with a special emphasis on safety. The DSMB has 3 members (1 biometrician, 2 clinical experts with experience in COVID-19, see appendix), who are independent of the medical institution of the coordinating investigators as well as of the different trial sites.

DSMB will monitor safety data after the first 20 patients and after the first 20 patients are enrolled in each arm, thereafter all 3 months. A special focus will be on potential side effects of the CP and camostat mesylate. Furthermore all 2-3 months, DSMB together with the principal coordinating investigators will review emerging alternative treatment options. Thus, DSMB will judge the information on efficacy and safety data in the context of newly emerging evidence for optimal treatment of COVID-19. The DSMB will provide recommendations to the sponsor including a recommendation to continue/discontinue the trial. DSMB Members are listed in the appendix.

9 QUALITY MANAGEMENT

9.1 Principal Investigator Responsibilities

Principal investigator responsibilities are set out in the German drug law (AMG), GCP-Verordnung (GCP-V), the ICH guideline for Good Clinical Practice (GCP) and in the Directive 2001/20/EC of the European Union.

The PI agrees to regular monitoring visits at the trial site and assures that the CRA will receive appropriate support in his activities. He is responsible for all data entered in the eCRFs. The PI will support sponsor audits and regulatory inspection(s) (e.g., BfArM, PEI, EMEA, local authorities) by providing direct access to the study facilities, to source documents, to CRFs, and to all other study-related documents.

9.2 Quality Assurance and Quality control

The sponsor assumes responsibility for implementing and maintaining quality assurance and quality control systems with written Standard Operation Procedures (SOPs) to ensure that the study is conducted and data are generated, documented and reported in compliance with the protocol, Good Clinical Practice, and the applicable regulatory requirements. The sponsor also takes responsibility for securing agreement from all involved parties to ensure direct access to the trial related sites, source data/documents, and reports for the purpose of monitoring and auditing by the sponsor and inspection by authorities. Quality control will be applied to each stage of the study to ensure that all data are reliable and have been processed correctly.

9.3 Study Monitoring

The trial site will be monitored by clinical research associates (CRA) of the KKSD to ensure the quality of the data collected. The objectives of the monitoring procedures are to ensure that the trial subject's safety and rights as a study participant are respected, that accurate, valid and complete data are collected, and that the trial is conducted in accordance with the trial protocol, the principles of GCP and local legislation.

A risk-based monitoring strategy will be implemented, where central monitoring will be combined with onsite-monitoring visits in order to achieve high protocol compliance and data quality, as well as to ensure patients` safety and rights.

According to a risk analysis, risk-bearing trial aspects will be monitored onsite. The CRA will check:

- Availability of the subject's informed consent

- Adherence to eligibility criteria
- Adherence to treatment according to the trial protocol
- Safety parameters
- Completeness of the trial documents at the trial sites
- Drug accountability

The PI, or a designated member of the trial staff, must be available at some time during monitoring visits to review data, resolve any queries and to allow direct access to the subject's records (e.g., medical records, office charts, hospital charts, and study related charts) for source data verification. The data collection must be completed prior to each visit and be made available to the inspecting representative so that the accuracy and completeness may be checked.

Aspects of trial conduct and any deficiencies found by the CRA will be discussed with the PI. The PI will reasonably consider corrective and preventive measures suggested by the CRA. The CRA confirms completed source data verification (SDV) by electronic signature in the eCRF.

9.4 Audits

Authorised representatives of the Sponsor may visit the trial sites to perform audits, including source data verification. The purpose of a Sponsor audit is to systematically and independently examine all study related activities and documents to determine whether these activities were conducted, and data were recorded, analysed, and accurately reported according to the protocol, Good Clinical Practice (GCP), guidelines of the International Conference on Harmonisation (ICH), and any applicable regulatory requirements.

9.5 Source Data and Patient Files

The information in original (source) documents and are defined as Source Data. They are kept in the patient files at the trial sites. The source documents should cover demographic and medical information, including laboratory data, medication, physical examination, etc.

9.6 Recording, Analyses and Reporting

The data management will follow a Remote Data Entry approach. All study data will be recorded in an Electronic Case Report Form (eCRF). The eCRF will be implemented in a Clinical Data Management System (CDMS) with Electronic Data Capture functionality (EDC).

Implementation of the eCRF will be performed by data managers from the KKSD in cooperation with the PCI and biostatisticians from the KKSD. The system complies with the relevant international regulations and standards and provides the capability to perform the major data management activities within a consistent, auditable and integrated electronical environment (query management, data entry, data validation). Range, validity and consistency checks will be implanted in the eCRF for application during data entry. There will be a comprehensive validation of the eCRF before release to the investigators. All data entry, modification or deletion will be recorded automatically in an audit trail indicating the original value, the new value, the reason for change, who made the change and when the change was made. A digital signature is implemented and included in the audit trail. The internet connection is secured by adequate technology. The computerized system is able to generate

accurate and complete copies of records in both hard copy and electronic form for copying, inspection and reviewing by regulatory authorities and Ethics Committees.

Data recorded by eCRF are pseudonymized. In the KKSD, data security and protection rules are strictly followed with the help of standard operating procedures and working instructions. The KKSD takes care that no unallowed access to the computer system takes place and that no data loss occurs by following adequate data backup procedures. Only specified persons involved in the trial get authorization to enter or access data in the clinical trial data base based on predefined roles.

9.7 Data Collection

All data will be entered in a central database via eCRF. As each study participant will get a unique subject number, no identification of subjects will be possible. Data corrections will be handled similar to new data entry. The audit trail of database ensures that changes in data can be followed by time point and identification of data manager.

Data recording for the individual patient starts as soon as the consent is signed and any study specific procedures are done for screening. Data is collected until the patient either terminates the study for any reason or after properly completing the follow-up phase after the treatment phase.

All data collected in the eCRF needs to be available as source data in the patient file as well. Care should be taken to collect data as completely as possible. All data should be recorded in a timely manner to ensure early quality checks and source data verification by the monitor.

The overall responsibility for data entered into the eCRF lies with the Principal Investigator at each site. The PI confirms correct data entry by electronic signature in the eCRF. The KKSD will supply the necessary documentation and training to the investigator, study site staff and study coordinator representatives, and will support the use of the eCRF through a help-desk with a ticketing system (support@kksd.de). During the trial, all findings will be documented on eCRF or other study specific paper forms by the responsible investigator or designated representatives. The investigator will maintain a list of these representatives. The data has to be complete, clear, accurate, legible and plausible. Missing examinations or dates have to be marked along with a justification/explanation.

The query management is performed electronically under the supervision of the monitor. Data corrections in the eCRF, if necessary, have to be performed by the investigator or designated representatives. Only these persons are allowed to the system and their identity during use will be registered. Data on patients collected on eCRF in the course of the trial will be documented in a pseudonymous fashion. For monitoring and auditing purposes, and to the greatest extent possible, all information must be traceable back to the source documents, which are generally maintained in the patient’s medical file. The data security is ensured by several measures. The source data remain in the respective hospital information system. The source documents, in particular documents with plain-text names of the participating patients, e.g. the patient identification list, are kept under strict control of the investigator at the trial site. They will not be transferred to the KKSD, the sponsor or other persons that were not authorized by the patient in the data protection declaration part of the informed consent form. More details regarding data management are outlined in the Data Management Manual.

10 STATISTICAL ANALYSIS

10.1 Statistical Hypotheses

The hypothesis to be investigated in the RES-Q HR study is that the early use of convalescent plasma (CP) or camostat mesylate reduces the likelihood of disease progression to WHO stages 4b-8 in SARS-CoV-2 positive, pre-existing patients at high risk for moderate or severe COVID-19.

In statistical terms, let Y_(i, k) be the primary outcome variable of patient i=1,2,…,n_k who is randomized to treatment k=CP, Camostat, SoC, placebo + SoC, where

$$Y_{i,k} = \begin{cases} 1 & \text{if WHO score is between 4b to 8} \\ 0 & \text{Otherwise} \end{cases}$$

Let $p_k = P(Y_{i,k} = 1)$ be the response rate for the treatment k. Then we operationalized the research hypothesis as

$$H_0: \begin{cases} p_{CP} & = & p_{SOC} \\ p_{Comostat} & = & p_{SOC+Placebo} \end{cases} vs, \quad H_1: \begin{cases} p_{CP} & \neq & p_{SOC} \\ p_{Comostat} & \neq & p_{SOC+Placebo} \end{cases}.$$

10.2 Sample Size Determination

Based on the evidence in the literature, it is assumed that 35% of patients in the target population will have disease progression towards WHO stages 4b-8.

In this study, the aim to prove that either the CP or camostat mesylate could have an improvement of 35% compare to their respective placebo groups. We also assume that a dropout rate of 5% is realistic under the context of the study.

When the sample size is 994 patients in total, each of the two-sample Z-tests for proportions with a 5% two-sided significance level will have 80% power to detect an OR of 0.547 assuming a dropout rate of 5 % and a randomization rate of two to one (2:1) in favor to treatment.

10.3 Populations for Analyses

The statistical analysis will be performed on the intention-to-treat (ITT) population, which includes all patients that have been randomized. The safety analysis will be based on the modified ITT population, where patients received at least one intervention (e.g. one infusion).

10.4 Statistical Analyses

10.4.1 Baseline descriptive statistics

Demographic and baseline data will be compared using appropriate statistical techniques. For continuous outcomes the mean and the standard deviation will be reported. The proportions in each category will describe categorical variables.

Descriptive statistics for the primary and secondary endpoints will be calculated for each treatment group. The summary results will be displayed using statistical graphics and tables.

10.4.2 Analysis of the Primary Efficacy Endpoint

Efficacy will be analyzed in the ITT (Intention-to-Treat) population. For the analysis of the primary endpoint, we plan to apply two-sample Z-test for proportions with a 5% two-sided

significance level. We will report the 95% confidence interval of the OR and rates differences δ between treatment and control groups. Given that these rates contrasts are pre-specified and they are design independent, no adjustments of statistical significance is required.

In addition to the classical statistical analysis, we plan to perform a Bayesian sensitivity analysis for the interpretation of the trial results. This analysis is based on the posterior probability of the OR and δ under a non-informative prior and a sceptical informative prior for the treatment effect.

If the amount of the missing data in the primary outcome variable exceeded the assumed dropout rate of 5%, the confirmatory analysis will be performed using a Bayesian analysis. Robustness of the trial conclusions will be assessed by calculating the posterior distribution of the ORs and δs under two different missing data assumptions: MAR (Missing at Random) and NMAR (Not Missing at Random).

10.4.3 Analysis of the Secondary Endpoint(s)

The secondary endpoints will be analyzed using descriptive statistics in a similar strategy as the primary endpoint. Further statistical details will be described in the SAP and in the final statistical report. The following secondary endpoints will be analyzed:

- Cumulative number of persons in the respective treatment arms versus SoC/placebo in WHO categories 4b-8 by day 8, day 14, day 56 and day 90
- Cumulative number of persons in the respective treatment arms versus SoC/placebo in WHO categories 3-4a by day 8, day 14, day 28, day 56 and day 90
- *"Event-free"* (not hospitalized, no COVID-19 associated long-term effects such secondary sclerosing cholangitis, pulmonary disease, no reinfection) survival at day 90 and evaluation of all-cause mortality at day 90 using Kaplan-Meier
- The proportion of patients with remdesivir therapy and WHO status at initiation of remdesivir
- The proportion of patients on dexamethasone therapy and WHO status at baseline dexamethasone
- Time to resolution of COVID-19 related symptoms (e.g. fever)
- Time to first negative SARS-CoV-2-PCR
- Duration of oxygen therapy (in days)
- Frequency of occurrence of COVID-19 pneumonia
- Percentage of participants in each group with need for mechanical ventilation/ECMO (and ventilation days)
- Duration of hospital stay (in days), duration in intensive care/IMC (in days)
- All-cause mortality at day 28
- Cumulative incidence of SAEs per group within 90 days follow up
- Cumulative incidence of grade 3/4 AEs per group within 90 days follow up
- SARS-CoV-2 antibody concentrations (IgA, IgG, NT) in serum on day 8, day 14, day 90
- Number of screening failures due to the lack of a suitable plasma preparation

10.4.4 Safety Analyses

The safety analysis will be performed on the modified ITT population, where patients received at least one intervention (e.g. one infusion, one dose of camostat mesylate).

Safety endpoints include death through day 28, SAEs, discontinuation of study infusions, and severe AEs. Each AE will be counted once for a given participant and graded by severity and relationship to COVID-19 or study intervention. AEs will be coded using the current version of the Medical Dictionary for Regulatory Activities (MedDRA). AEs will be presented by system organ class, duration (in days), start-date and stop-date. Adverse events leading to premature discontinuation from the study intervention and serious treatment-emergent AEs should be presented either in a table or in a listing.

10.4.5 Planned Interim Analyses

10.4.5.1 Interim Analyses

A data and safety monitoring board (DSMB) will monitor ongoing results to ensure patient well-being and safety as well as study integrity. The DSMB will be asked to recommend early termination or trial modifications only when there is clear and substantial evidence of a treatment difference. More details about the interim report is described in a separate guidance document for the DSMB.

10.4.5.2 Interim Efficacy Review

We plan an interim analysis for efficacy when 50% of the participants have been randomized. We use a two sided O'Brien-Fleming spending function at a statistical significant level of 5%. The following R output shows the numerical results upper and lower bounds:

```
## Overall alpha:  0.05
##
## Type: Two-Sided Symmetric Bounds
## Lower alpha:  0.025
## Upper alpha:  0.025
## Spending function:  O'Brien-Fleming
##
## Boundaries:
##    Time    Lower   Upper   Exit pr.  Diff. pr.
## 1   0.5  -2.9626  2.9626  0.0030506  0.0030506
## 2   1.0  -1.9686  1.9686  0.0500000  0.0469494
```

In addition to the classical statistical interim analysis based on the O'Brien-Fleming spending function; we plan to report to the DSMB a Bayesian sensitivity analysis based on the posterior distribution of the OR and δ under a sceptical informative prior (38, page 159 and page 206). This sceptical prior is constructed by assuming that the probability that the treatment effect is as large as the alternative hypothesis is only 1%. The resulting posterior distributions of OR and δ will be summarized by the posterior means, standard deviation, and quantiles of 2.5%, 25%, 50%, 75% and 95.75%.

The DSMB reports will contain statistical technical details and results interpretation. These results may help to stop the trial for early efficacy or futility. The unblinded statistical team will prepare these closed reports for DSMB.

10.4.6 Sub-Group Analyses

Subgroups to be analyzed for primary and secondary endpoints are as follow:

- According to age group
- According to concomitant number of disease and type of disease
- According to BMI

10.4.7 Exploratory Analyses

Not applicable.

10.5 Reporting

10.5.1 Statistical Report

Statistical technical details will be documented in the statistical analysis plan (SAP) before the study database lock. The SAP will be developed in cooperation with the Sponsor and the Principal Investigator.

Statistical documentation including the SAP, the DSMB reports and the final statistical report will be develop using dynamic generating reporting tools that validate replicability of statistical results (*Markdown*, *knitr*, etc.).

Statistical computations will be implemented using the statistical language R (39) and Bayesian computations, e.g. Markov Chain Monte Carlo, will be implemented using JAGS (40). The validation of the scripts used for analyses will be report with as part of the study documentation.

10.5.2 Final Report

Competent authorities and the Ethics Committee will be informed within 90 days of the completion of the trial. The composition of a final integrated report will be conducted in accordance with ICH E3 "Structure and Contents of Clinical Study Reports". It will be submitted to BfArM, PEI, and Ethics Committee within one year of the completion of the clinical trial. The DSMB will also receive the final report. Furthermore, the results will be published online at EU Clinical Trials Register in accordance to EU legislation.

10.5.3 Publication Policy

The results obtained will be communicated to the COVID-19 treating centers regardless of the outcome of the study and will help to improve the treatment of these patients. The results will be published in medical journals and communicated at scientific/clinical meetings and similar events. In addition to scientific findings, the study could contribute to the development of therapy standards and will be communicated in the respective associations/societies (i.e. DGI, STAKOP). Furthermore, the outpatient approach could lead to savings in the health care system. In addition, the tested approach could possibly also be used as chemoprophylaxis in high-risk groups after a corresponding COVID-19 contact and thus provide the basis for further studies.

11 ETHICAL, LEGAL AND REGULATORY ASPECTS

11.1 ICH-GCP-Guidelines

This trial will be conducted in accordance with the current ICH-GCP-guidelines. Good Clinical Practice (ICH-GCP (E6(R2)) is an international ethical and scientific quality standard for designing, conducting, recording and reporting trials that involve the participation of human subjects. Compliance with this standard provides public assurance that the rights, safety and well-being of trial subjects are protected, consistent with the principles that have their origin in the Declaration of Helsinki, and that the clinical trial data are credible.

11.2 Legal Requirements of the Study

11.2.1 Approval of Ethics Committee

The trial will not be initiated before the protocol, the informed consent and the patient information form have been reviewed and received approval from the Independent Ethics Committee (IEC) responsible for the coordinating investigator's site and all responsible Ethic Committees in every participating trial site (Ethics Committee of the Medical Faculty of Heinrich-Heine University). Should a protocol amendment be made that needs IEC approval (i.e., a substantial amendment), the changes in the protocol will not be instituted until the amendment and revised informed consent (if appropriate) have been reviewed and received approval from the responsible IEC. A protocol amendment intended to eliminate an apparent immediate hazard to patients may be implemented immediately providing that the appropriate regulatory authorities and IEC are notified as soon as possible and an approval is requested. Protocol amendments only for logistical or administrative changes may be implemented immediately, but the IEC needs to be informed as well.

The Ethics Committee will also be informed of all suspected SUSARs and of regular or premature termination of the study.

11.2.2 Approval of national competent Authorities

The trial will be submitted to the relevant national competent authorities (Bundesinstitut für Arzneimittel und Medizinprodukte BfArM, and Paul-Ehrlich-Institut PEI) for approval. The trial will only start after approvals have been granted. Any substantial amendments to the protocol will be submitted as well, if applicable according to German law.

11.2.3 Notification to Local Authorities

The relevant local authorities will be informed about start, termination, and, if applicable, about suspension and interruption of the clinical trial.

11.3 Patient Information and Informed Consent

Before enrolment every patient will receive full oral and written information about the nature, purpose, expected advantages and possible risks of the trial.

The patient will agree to participation in the trial by signing the informed consent form. Patients must be given an opportunity to enquire about details of the study. After a sufficient period of time for the individual's consideration and decision, comprehension and consent shall be documented on the consent form by the dated signature of the patient and the

Investigator. If a patient is able to consent but cannot sign himself/herself, oral information and written consent need to be testified and signed by a witness.

Documentation that informed consent occurred prior to the subject's entry into the study, and the informed consent process should be recorded in the subject's source documents. The fully signed and dated informed consent form must be maintained in the investigator site file. A copy is handed to the patient.

11.4 Patient Insurance

According to §40 Article 1 No. 8 and Article 3 AMG (German Drug Law) the Sponsor of the study must obtain insurance coverage for eventually occurring damage caused by the treatment or any actions taken according to the treatment plan.

11.5 Data Privacy and Confidentiality

The provisions of data protection legislation will be observed. It is assured by the sponsor that all investigational materials and data will be pseudonymised in accordance with data protection legislation.

Trial participants will be informed that their pseudonymised data will be handled in accordance with applicable law. Subjects who do not agree to data handling as described in the informed consent form will not be enrolled into the trial.

11.6 Archiving of Data / Access to Records

Originals of all study-related report forms and original data / medical records of study participants will be stored in the study headquarters at the trial site for at least 10 years after completion of the trial (§13(10) GCP-V). German transfusion law requires storage up to 30 years (§14 (3) TFG).

The investigator / principle investigator stores all administrative documents (correspondence with the ethics committee, local and competent authorities, trial centres, study sites), patient identification log, the signed patient consent forms, copies of the data documentation form and common study documentation (protocol, amendments) for the duration mentioned above. A list allowing patient identification will be kept for 30 years (directive 2001/83/EG).

12 FINANCING

The trial is financed by the German ministry of health (Bundesministerium für Gesundheit BMG).

13 REFERENCES

1. Yang X, Yu Y, Xu J, Shu H, Xia J, Liu H, et al. Clinical course and outcomes of critically ill patients with SARS-CoV-2 pneumonia in Wuhan, China: a single-centered, retrospective, observational study. Lancet Respir Med. 2020;8(5):475-81.
2. Zhu N, Zhang D, Wang W, Li X, Yang B, Song J, et al. A Novel Coronavirus from Patients with Pneumonia in China, 2019. N Engl J Med. 2020;382(8):727-33.
3. Russell TW, Hellewell J, Jarvis CI, van Zandvoort K, Abbott S, Ratnayake R, et al. Estimating the infection and case fatality ratio for coronavirus disease (COVID-19) using age-adjusted data from the outbreak on the Diamond Princess cruise ship, February 2020. Euro Surveill. 2020;25(12).
4. Pastor-Barriuso R, Perez-Gomez B, Hernan MA, Perez-Olmeda M, Yotti R, Oteo J, et al. Infection fatality risk for SARS-CoV-2: a nationwide seroepidemiological study in the non-institutionalized population of Spain. medRxiv. 2020:2020.08.06.20169722.
5. Ward H, Atchison CJ, Whitaker M, Ainslie KEC, Elliott J, Okell LC, et al. Antibody prevalence for SARS-CoV-2 in England following first peak of the pandemic: REACT2 study in 100,000 adults. medRxiv. 2020:2020.08.12.20173690.
6. Poletti P, Tirani M, Cereda D, Trentini F, Guzzetta G, Marziano V, et al. Age-specific SARS-CoV-2 infection fatality ratio and associated risk factors, Italy, February to April 2020. Euro Surveill. 2020;25(31).
7. Giangreco G. Case fatality rate analysis of Italian COVID-19 outbreak. J Med Virol. 2020;92(7):919-23.
8. Karagiannidis C, Mostert C, Hentschker C, Voshaar T, Malzahn J, Schillinger G, et al. Case characteristics, resource use, and outcomes of 10 021 patients with COVID-19 admitted to 920 German hospitals: an observational study. Lancet Respir Med. 2020.
9. Moon AM, Webb GJ, Aloman C, Armstrong MJ, Cargill T, Dhanasekaran R, et al. High mortality rates for SARS-CoV-2 infection in patients with pre-existing chronic liver disease and cirrhosis: Preliminary results from an international registry. J Hepatol. 2020;73(3):705-8.
10. Petrilli CM, Jones SA, Yang J, Rajagopalan H, O'Donnell L, Chernyak Y, et al. Factors associated with hospital admission and critical illness among 5279 people with coronavirus disease 2019 in New York City: prospective cohort study. BMJ. 2020;369:m1966.
11. Docherty AB, Harrison EM, Green CA, Hardwick HE, Pius R, Norman L, et al. Features of 20 133 UK patients in hospital with covid-19 using the ISARIC WHO Clinical Characterisation Protocol: prospective observational cohort study. BMJ. 2020;369:m1985.
12. Jain V, Yuan JM. Predictive symptoms and comorbidities for severe COVID-19 and intensive care unit admission: a systematic review and meta-analysis. Int J Public Health. 2020;65(5):533-46.
13. Roncon L, Zuin M, Rigatelli G, Zuliani G. Diabetic patients with COVID-19 infection are at higher risk of ICU admission and poor short-term outcome. J Clin Virol. 2020;127:104354.
14. ElGohary GM, Hashmi S, Styczynski J, Kharfan-Dabaja MA, Alblooshi RM, de la Camara R, et al. The risk and prognosis of COVID-19 infection in cancer patients: A systematic review and meta-analysis. Hematol Oncol Stem Cell Ther. 2020.
15. Grein J, Ohmagari N, Shin D, Diaz G, Asperges E, Castagna A, et al. Compassionate Use of Remdesivir for Patients with Severe Covid-19. N Engl J Med. 2020;382(24):2327-36.
16. Wang Y, Zhang D, Du G, Du R, Zhao J, Jin Y, et al. Remdesivir in adults with severe COVID-19: a randomised, double-blind, placebo-controlled, multicentre trial. Lancet. 2020;395(10236):1569-78.
17. Mair-Jenkins J, Saavedra-Campos M, Baillie JK, Cleary P, Khaw FM, Lim WS, et al. The effectiveness of convalescent plasma and hyperimmune immunoglobulin for the treatment of severe acute respiratory infections of viral etiology: a systematic review and exploratory meta-analysis. J Infect Dis. 2015;211(1):80-90.
18. Cheng Y, Wong R, Soo YO, Wong WS, Lee CK, Ng MH, et al. Use of convalescent plasma therapy in SARS patients in Hong Kong. Eur J Clin Microbiol Infect Dis. 2005;24(1):44-6.

19. Joyner MJ, Senefeld JW, Klassen SA, Mills JR, Johnson PW, Theel ES, et al. Effect of Convalescent Plasma on Mortality among Hospitalized Patients with COVID-19: Initial Three-Month Experience. medRxiv. 2020.
20. Li L, Zhang W, Hu Y, Tong X, Zheng S, Yang J, et al. Effect of Convalescent Plasma Therapy on Time to Clinical Improvement in Patients With Severe and Life-threatening COVID-19: A Randomized Clinical Trial. JAMA. 2020;324(5):460-70.
21. Hoffmann M, Kleine-Weber H, Schroeder S, Kruger N, Herrler T, Erichsen S, et al. SARS-CoV-2 Cell Entry Depends on ACE2 and TMPRSS2 and Is Blocked by a Clinically Proven Protease Inhibitor. Cell. 2020;181(2):271-80 e8.
22. Beard KR, Brendish NJ, Clark TW. Treatment of influenza with neuraminidase inhibitors. Curr Opin Infect Dis. 2018;31(6):514-9.
23. Bloch EM, Shoham S, Casadevall A, Sachais BS, Shaz B, Winters JL, et al. Deployment of convalescent plasma for the prevention and treatment of COVID-19. The Journal of clinical investigation. 2020;130(6):2757-65.
24. Abolghasemi H, Eshghi P, Cheraghali AM, Imani Fooladi AA, Bolouki Moghaddam F, Imanizadeh S, et al. Clinical efficacy of convalescent plasma for treatment of COVID-19 infections: Results of a multicenter clinical study. Transfus Apher Sci. 2020:102875.
25. Okba NMA, Muller MA, Li W, Wang C, GeurtsvanKessel CH, Corman VM, et al. Severe Acute Respiratory Syndrome Coronavirus 2-Specific Antibody Responses in Coronavirus Disease Patients. Emerg Infect Dis. 2020;26(7):1478-88.
26. Rojas M, Rodriguez Y, Monsalve DM, Acosta-Ampudia Y, Camacho B, Gallo JE, et al. Convalescent plasma in Covid-19: Possible mechanisms of action. Autoimmun Rev. 2020;19(7):102554.
27. Xia X, Li K, Wu L, Wang Z, Zhu M, Huang B, et al. Improved clinical symptoms and mortality among patients with severe or critical COVID-19 after convalescent plasma transfusion. Blood. 2020;136(6):755-9.
28. Yan R, Zhang Y, Li Y, Xia L, Guo Y, Zhou Q. Structural basis for the recognition of SARS-CoV-2 by full-length human ACE2. Science. 2020;367(6485):1444-8.
29. Zhou Y, Vedantham P, Lu K, Agudelo J, Carrion R, Jr., Nunneley JW, et al. Protease inhibitors targeting coronavirus and filovirus entry. Antiviral Res. 2015;116:76-84.
30. Joyner MJ, Bruno KA, Klassen SA, Kunze KL, Johnson PW, Lesser ER, et al. Safety Update: COVID-19 Convalescent Plasma in 20,000 Hospitalized Patients. Mayo Clin Proc. 2020;95(9):1888-97.
31. Arvin AM, Fink K, Schmid MA, Cathcart A, Spreafico R, Havenar-Daughton C, et al. A perspective on potential antibody-dependent enhancement of SARS-CoV-2. Nature. 2020;584(7821):353-63.
32. Eroshenko N, Gill T, Keaveney MK, Church GM, Trevejo JM, Rajaniemi H. Implications of antibody-dependent enhancement of infection for SARS-CoV-2 countermeasures. Nat Biotechnol. 2020;38(7):789-91.
33. Mankarious S, Lee M, Fischer S, Pyun KH, Ochs HD, Oxelius VA, et al. The half-lives of IgG subclasses and specific antibodies in patients with primary immunodeficiency who are receiving intravenously administered immunoglobulin. J Lab Clin Med. 1988;112(5):634-40.
34. Duan K, Liu B, Li C, Zhang H, Yu T, Qu J, et al. Effectiveness of convalescent plasma therapy in severe COVID-19 patients. Proceedings of the National Academy of Sciences of the United States of America. 2020;117(17):9490-6.
35. An EU programme of COVID-19 convalescent plasma collection and transfusion 2020 [Available from: https://ec.europa.eu/health/sites/health/files/blood_tissues_organs/docs/guidance_plasma_covid19_en.pdf.
36. Kawase M, Shirato K, van der Hoek L, Taguchi F, Matsuyama S. Simultaneous treatment of human bronchial epithelial cells with serine and cysteine protease inhibitors prevents severe acute respiratory syndrome coronavirus entry. J Virol. 2012;86(12):6537-45.
37. Tamura Y, Hirado M, Okamura K, Minato Y, Fujii S. Synthetic inhibitors of trypsin, plasmin, kallikrein, thrombin, C1r-, and C1 esterase. Biochim Biophys Acta. 1977;484(2):417-22.

38. Spiegelhalter DJ, Abrams MP, Myles JP. Bayesian approaches to clinical trials and health-care evaluation Wiley & Sons Ltd.; 2004.
39. Team RC. R: A language and environment for statistical computing Vienna, Austria2019 [Available from: https://www.R-project.org/.
40. Plummer M, editor JAGS: A Program for Analysis of Bayesian Graphical Models Using Gibbs Sampling. 3rd International Workshop on Distributed Statistical Computing (DSC 2003); 2003; Vienna, Austria.

14 APPENDICES

14.1 Schedule of Events

	Screening	Day 1 Baseline	Day 3	Day 5	Day 8 EOT	Day 14 Follow-up	Day 28 Follow-up ± 5 days, tel	Day 56 Follow-up ± 5 days, tel	Day 90 Follow-up ± 5 days
Informed Consent	X								
Eligibility Assessment	X								
Pregnancy Test	X				X				
Demographics	X								
Medical History	X								
Concomitant Medications	X	X	X	X	X	X	X	X	X
Physical Examination	X	X	X	X	X	X			X
ECG	X				X				
Height	X								
Weight	X								
Vital Signs[1]	X	X	X	X	X	X			X
Hematology (CBC, differential)	X		X	X	X	X			X
Chemistry (Na, K, glucose, Crea, BUN, GOT, GPT, GGT, ALP, Bilirubin, PTT, INR, CRP, PCT, CK, NT-proBNP, D-Dimers, ferritin, fibrinogen, Troponin)	X		X	X	X	X			X
Blood group	X								
Anti-HIV, anti-HCV, HBs AG, anti-HBc, anti-HEV	X								X
IL-6	X		X	X	X	X			X
N/P swab, SARS-CoV-2 PCR	X		X	X	X	X			X

Anti-SARS-CoV-2 antibody titers	X		X	X	X	X			X
Optional: Full blood/ serum / plasma/ urine for exploratory biomarkers, plasma levels camostat	X		X	X	X	X			X
Respiratory Status [2]	X	X	X	X	X	X			X
8-point Ordinal Scale	X	X	X	X	X	X	X	X	X
Administer IMP (plasma)		X	X[3]						
Administer IMP (Camostat mesylate / placebo)		X[4]	X[4]	X[4]					
SF-12 Quality of Life Questionnaire, VAS	X						X		X
Adverse Events	X	X	X	X	X	X	X	X	X

1) Vital signs: Includes heart rate, respiratory rate, temperature, blood pressure, SpO_2
2) Respiratory status: 1-ambiant air, 2-O2 supplementation nasal mask < 4l/min, 3- O2 supplementation mask > 4 l/min, 4-NIV/high flow, 5- intubation and mechanical ventilation, 6- ECMO
3) Administer 3rd unit of CP if patient weighs ≥ 150 kg
4) Three times daily 200 mg, day 1 - 7

14.2 Visual Analogue Scale (VAS)

Um Sie bei der Einschätzung, wie gut oder wie schlecht Ihr Gesundheitszustand ist, zu unterstützen, haben wir eine Skala gezeichnet, ähnlich einem Thermometer. Der beste denkbare Gesundheitszustand ist mit "100" gekennzeichnet, der schlechteste mit "0".

Wir möchten Sie nun bitten, auf dieser Skala zu kennzeichnen, wie gut oder schlecht Ihrer Ansicht nach Ihr persönlicher Gesundheitszustand heute ist. Bitte verbinden Sie dazu den untenstehenden Kasten mit dem Punkt auf der Skala, der Ihren heutigen Gesundheitszustand am besten wiedergibt.

Ihr heutiger Gesundheitszustand

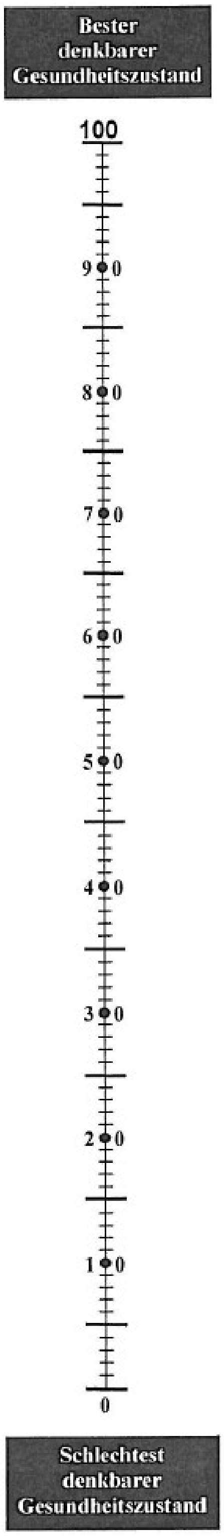

14.3 Data Safety and Monitoring Board DSMB

Separate document: RES-Q HR DSMB members

14.4 Quality of life questionnaire (SF-12)

Separate document
